# Hepatocellular Carcinoma Immune Landscape and the Potential of Immunotherapies

**DOI:** 10.3389/fimmu.2021.655697

**Published:** 2021-03-18

**Authors:** Julie Giraud, Domitille Chalopin, Jean-Frédéric Blanc, Maya Saleh

**Affiliations:** ^1^University of Bordeaux, CNRS, ImmunoConcEpT, UMR 5164, Bordeaux, France; ^2^University of Bordeaux, INSERM UMR 1053, Bordeaux, France; ^3^Department of Oncology, CHU Bordeaux, Haut Leveque Hospital, Pessac, France; ^4^Department of Medicine, McGill University, Montreal, QC, Canada

**Keywords:** immunotherapy, immune checkpoint inhibitors, tumor microenvironment, tumor-associated macrophages, immunosuppression, inflammation, cirrhosis, NASH

## Abstract

Hepatocellular carcinoma (HCC) is the most common liver tumor and among the deadliest cancers worldwide. Advanced HCC overall survival is meager and has not improved over the last decade despite approval of several tyrosine kinase inhibitors (TKi) for first and second-line treatments. The recent approval of immune checkpoint inhibitors (ICI) has revolutionized HCC palliative care. Unfortunately, the majority of HCC patients fail to respond to these therapies. Here, we elaborate on the immune landscapes of the normal and cirrhotic livers and of the unique HCC tumor microenvironment. We describe the molecular and immunological classifications of HCC, discuss the role of specific immune cell subsets in this cancer, with a focus on myeloid cells and pathways in anti-tumor immunity, tumor promotion and immune evasion. We also describe the challenges and opportunities of immunotherapies in HCC and discuss new avenues based on harnessing the anti-tumor activity of myeloid, NK and γδ T cells, vaccines, chimeric antigen receptors (CAR)-T or -NK cells, oncolytic viruses, and combination therapies.

## Preface

The liver is a critical hub of metabolism, glucose storage, lipid and cholesterol homeostasis, detoxification and processing of xenobiotics, endocrine regulation of growth signaling, blood volume regulation, and immune surveillance. These essential functions are coordinated by multiple cell types: the hepatocytes, which make up 80% of the liver volume; the cholangiocytes, which line the biliary ducts and are the second most abundant parenchymal cells of the liver; the liver sinusoidal endothelial cells (LSECs), which line the hepatic sinusoidal walls and display specialized functions in scavenging, antigen presentation and leukocyte recruitment [reviewed in (1)]; the hepatic stellate cells (HSCs), the body's largest storage site of vitamin A at quiescent state; and the liver-resident immune cells, which are particularly enriched in this important immune organ. The liver is continuously challenged with microbial- and danger-associated molecular patterns (MAMPs and DAMPs) and non-self-peptides derived from dietary and gut-derived microbial antigens. Its capacity to deal with these insults is reflected by its particular immune environment. Indeed, the liver hosts the largest population of tissue-resident macrophages, known as Kupffer cells (KCs). It also exhibits a high frequency of tissue-resident lymphocytes, namely natural killer (NK) cells, NKT cells, conventional αβ T cells, unconventional γδ T cells and B cells. The liver's diverse immunotolerance mechanisms limit the development of chronic liver diseases, including cirrhosis and liver cancers.

**Figure 1:**
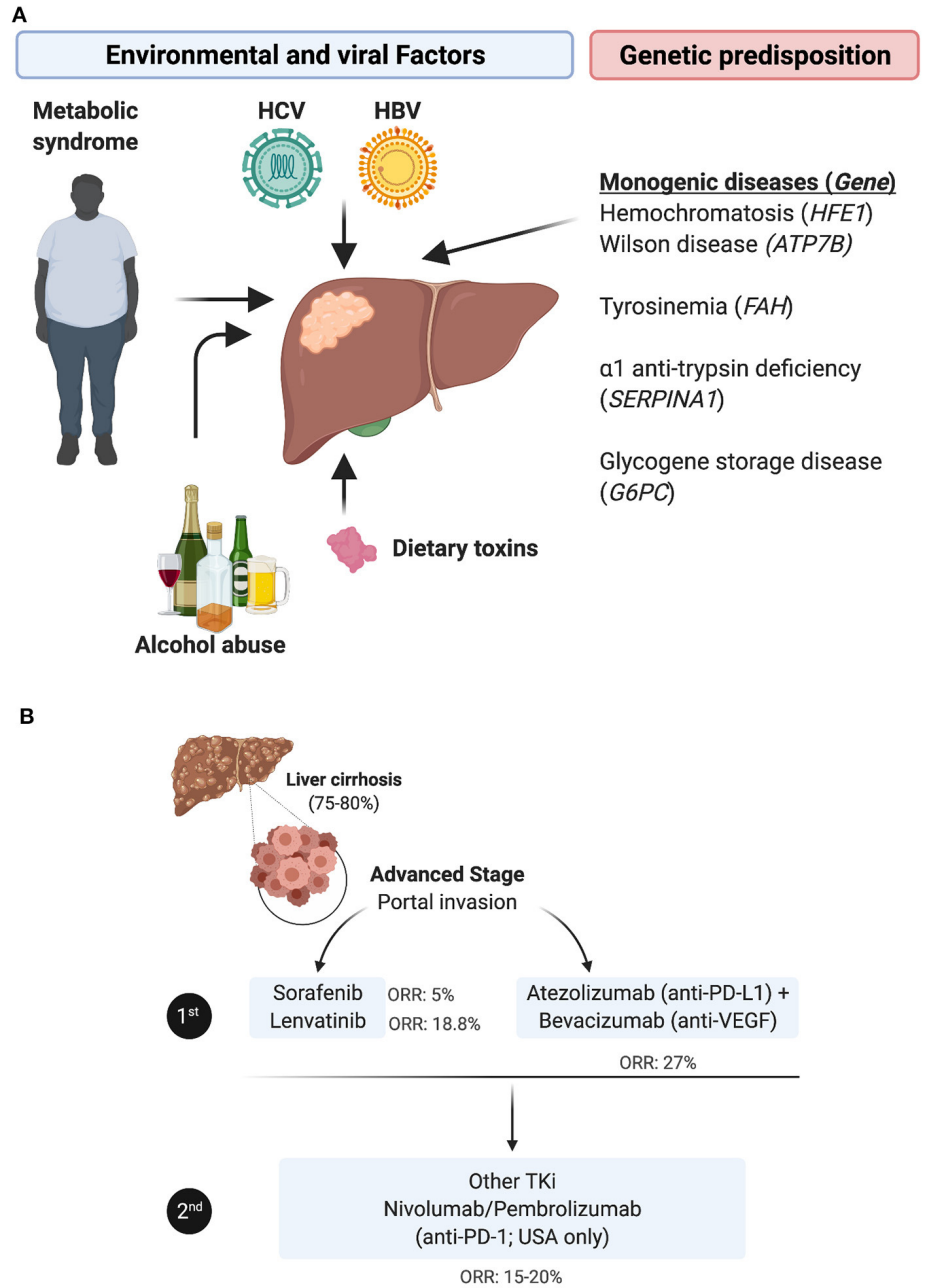
HCC etiologies, genetic predisposition and current standard of care for the advanced stage. **(A)** HCC etiologies include chronic infection with HBV or HCV, alcohol abuse, dietary toxins and/or the metabolic syndrome linked to obesity and type 2 diabetes. In rare cases, HCC stems from a monogenic disease e.g., hemochromatosis, caused by mutations in the homeostatic iron regulator gene *HFE1*; Wilson disease involving mutations in the ATPase copper transporting beta gene *ATP7B*; tyrosinemia, resulting from mutations in the gene encoding fumarylacetoacetate hydrolase *FAH*, α1-trypsin deficiency caused by mutations in serpin family A member 1 *SERPINA1;* or glycogen storage disease, in which the glucose-6-phosphatase gene is mutated. **(B)** The standard of care for treating patients with advanced HCC has been revised with the approval of immune checkpoint inhibitors. In first line, patients are administered TKi, mainly sorafenib or lenvatinib, or given the newly approved combination of bevacizumab (anti-VEGF) + atezolizumab (anti-PD-L1). In second line, patients refractory to TKi are treated with other TKIs, whereas anti-PD-1 ICI, nivolumab or pembrolizumab, have only been approved in the USA as an option for second line (despite the lack of superior efficacity in phase III trials compared to TKi).

Hepatocellular carcinoma (HCC), which accounts for approximately 90% of the incidence of all primary liver cancers, is the 5th most prevalent cancer worldwide and the 4th leading cause of death globally (2). Both environmental and genetic risk factors contribute to the etiology of HCC. The most notable environmental and potentially preventable risk factors include oncogenic virus infection with hepatitis B virus (HBV) or hepatitis C virus (HCV), alcohol abuse, and the metabolic syndrome related to obesity and diabetes mellitus [reviewed in (3)]. In addition, some rare monogenic diseases and several single nucleotide polymorphisms (SNPs) predispose individuals to HCC [reviewed in (4)] (Figure 1A). HCC incidence has doubled in the last three decades in the US, presumably due to high prevalence of HCV infection in the mid 1900's and increasing obesity-related non-alcoholic fatty liver disease (NAFLD) progressing to non-alcoholic steatohepatitis (NASH). Accordingly, suppression of HBV/HCV infections may improve HCC clinical outcomes, but few patients with HCC are cured of their hepatic infections due to treatment cost, compliance and toxicity issues, and NAFLD is expected to become the major risk factor for developing HCC in developed countries in the near future (5). In very early or early-stage HCC (stage 0/A, according to the Barcelona Clinic Liver Cancer [BCLC] staging system), the most effective therapeutic option remains surgical resection, liver transplantation or percutaneous local ablation. In this early stage, the median overall survival (mOS) is >60 months with a 5-year survival of 60–80%, but the 5-year recurrence rate is up to 70% [reviewed in (6)]. However, the large majority of HCCs are diagnosed at an intermediate (stage B) or an advanced stage (stage C), when the mOS is ~11–20 months with a 5-year survival of 16%.

The therapeutic options for these stages are limited to locoregional treatments, including transarterial chemoembolization (TACE) or radioembolization with yttrium 90 (90Y)-microspheres, and systemic treatment with multi tyrosine kinase inhibitors (TKi), such as Sorafenib (7) or Lenvatinib (8), according to international guidelines (9). While approved as a first-line therapy, these TKi improve mOS by 3 months (7, 8, 10) and are associated with significant side effects (11). In patients that progress following first line TKi treatment, the second-line options have been, until recently, alternative TKi, primarily regorafenib (12) and cabozantinib (13), or the fully human monoclonal antibody targeting vascular-endothelial growth factor (VEGF) receptor type 2 (VEGF-R2) ramucirumab (14). More recently, immune checkpoint inhibitors (ICI) have emerged as an alternative therapy in HCC and two anti-PD-1 drugs, nivolumab and pembrolizumab, have been approved in the USA based on two trials (15, 16) as a second line treatment for patients with advanced HCC refractory to sorafenib. The overall response rate (ORR) of nivolumab was reported to be 23% in sorafenib-naïve patients and 16-19% in sorafenib-experienced patients, with a mOS of 15 months. However, this did not reproduce in the phase III trial checkmate 459, in which the ORR to nivolumab in sorafenib-naïve patients was 15%, with a mOS of 16 months, i.e., not different from that with sorafenib. Further, in a recent trial, pembrolizumab monotherapy did not statistically impact HCC patients mOS and progression-free survival (PFS), as a second-line treatment (17). The combination of Regorafenib (angiogenesis inhibitor) and nivolumab has next been proposed as a second line treatment in sorafenib non-responders. This year, the combination of atezolizumab (anti-PD-L1) and bevacizumab (anti-VEGF) has obtained approval as a new first line therapy, as it improved mOS > 17 months (18) (Figure 1B). However, despite this therapeutic advance, ~75% of HCC patients do not respond to these immunotherapies for unclear reasons. While there is evidence that boosting the activity of tumor-specific T cells might benefit patients with HCC, the underlying chronic inflammation renders this cancer's tumor microenvironment (TME) somewhat unique, and highlights the urgent need to further explore this organ-specific immunity, identify biomarkers to select patients who are likely to respond to such treatments, and develop new immunotherapies combinations.

## The Landscape of Parenchymal, Stromal and Immune Cells in the Healthy vs. Cirrhotic Liver

**Figure 2:**
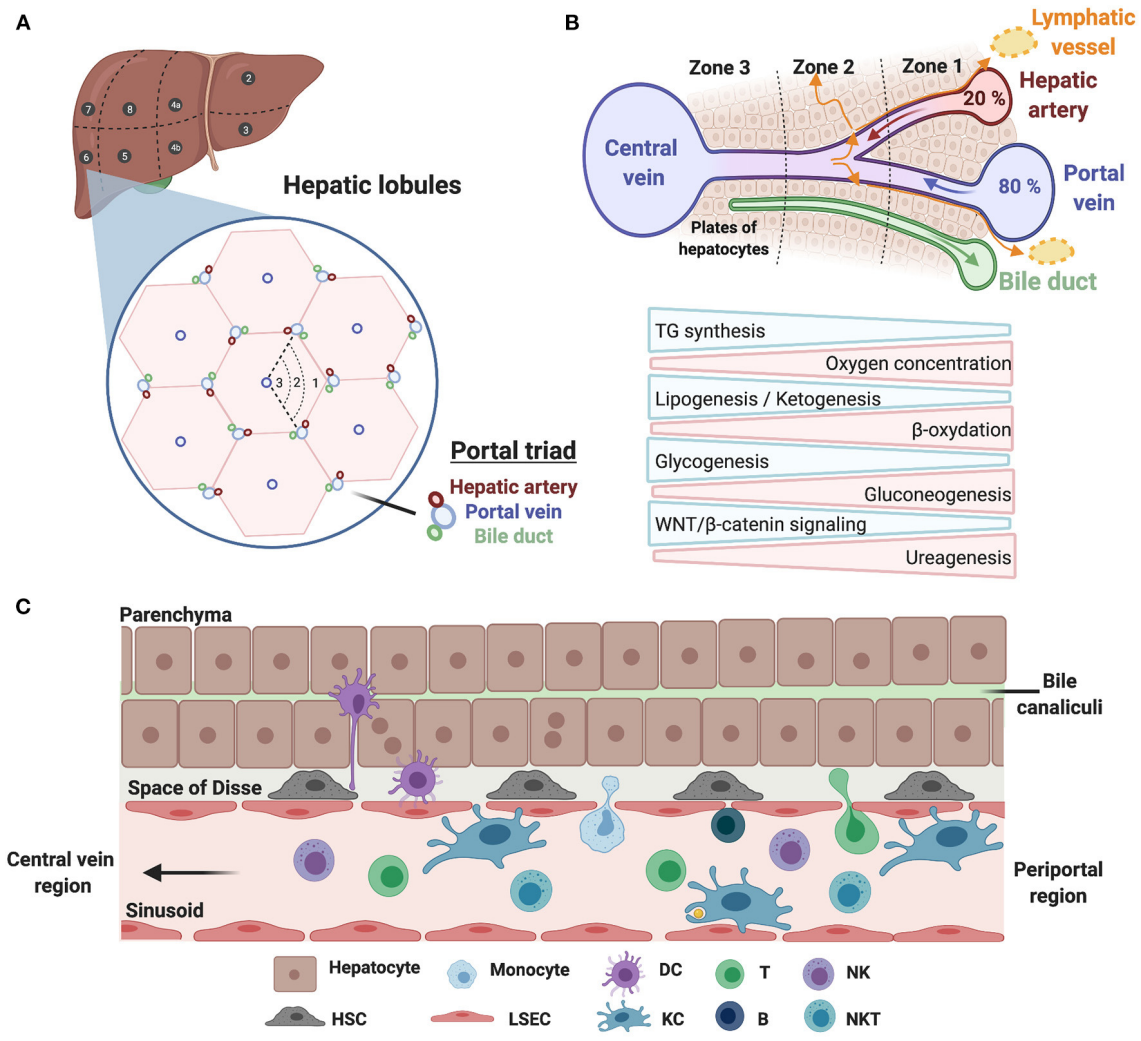
Architecture of the human liver and its immune system. **(A)** Schematic illustration of the human liver anatomy namely its 8 segments, hepatic lobules, and triads of portal vein/hepatic artery/bile duct. **(B)** The liver zonation. Oxygen and metabolic gradients define three liver zones with specialized hepatocytes functions. **(C)** A zoom on hepatic cellular interactions across the sinusoids, the space of Disse and the hepatocyte plates. Liver sinusoidal endothelial cells (LSECs) line the liver sinusoid by forming a fenestrated monolayer. Their basal side interacts with hepatocytes and hepatic stellate cells (HSCs) in the space of Disse, whereas their luminal side interacts with liver-resident leukocytes, including Kupffer cells (KCs).

Prior to delving into the immune landscape and immunosuppressive mechanisms of HCC, we briefly overview the architecture of the liver and its immune system under physiological conditions, and highlight specific changes occurring in cirrhosis. Anatomically, the human liver is composed of eight functional segments organized into hepatic lobules containing their portal vein, hepatic artery and bile duct triads (Figure 2A). Around 80% of the blood supply is delivered from the gut via the portal vein, while the remaining 20% flows through the hepatic artery. Upon mixing, the blood equilibrates and drains across the lobule through the hepatic sinusoids into the central veins, while the bile flows in the opposite direction via bile canaliculi. Such an organization creates oxygen and metabolic gradients, referred to as liver zonation, controlled in part by WNT/β-catenin signaling (Figure 2B). Liver sinusoids are lined by a fenestrated monolayer of LSECs that lack a basement membrane, allowing the blood to directly reach the underlying hepatocytes, organized in two-layered plates. The luminal side of LSECs interacts with liver resident immune cells, such as KCs, whereas their basal side, facing the space of Disse, interacts with hepatocytes and HSCs (Figure 2C). The liver has long been considered as a site of immune tolerance. This was based on early findings that transplanted allogeneic liver was significantly better tolerated than other organs, and patients required low levels of immunosuppression [reviewed in (19)]. Liver immune tolerance stems from complex interactions among liver-resident cells and peripheral leukocytes, and involves poor or incomplete activation of CD4^+^ and CD8^+^ T cells, elevated expression of immune checkpoints and an immunosuppressive environment mediated by IL-10 and TGFβ [reviewed in (20)]. KCs that function to preserve tissue homeostasis through their phagocytic and antigen presentation activity are important players in maintaining immune tolerance. Interestingly, a recent paper from the Germain group unraveled that microbiota sensing by LSECs imposes a chemokine gradient around the portal triads resulting in discriminate abundance of KCs and other immune cells (e.g., NKT cells) in periportal regions. Functionally, such an “immune zonation” is critical in limiting local infection and associated inflammatory tissue damage and in preventing the systemic spread of bacteria (21). Besides KCs, hepatic NK cells are capable of directly killing stressed cells, and mediate antibody-dependent cell cytotoxicity (ADCC) upon engagement of CD16 (FcγRIIIA). Their activity is regulated by a dynamic equilibrium between activating [NKG2D, NKp46 (NCR1), NKp44 (NCR2), and NKp30 (NCR3)] and inhibitory (KIR and NKG2A) receptors. In addition to producing various cytokines, chemokines, and growth factors, they maintain immune tolerance through expression of immune checkpoints. Liver-resident NK cells (LrNK) differ from conventional NK (cNK) cells with respect to their origin, phenotypes and functions. Notably, LrNK cells share functional properties with innate lymphoid cells (ILCs) commonly found in mucosal tissues. NKT cells, which also express the NK cell marker CD56, actively patrol the liver and contribute to the clearance of pre-malignant senescent hepatocytes (22). They are recruited via the chemokine receptor CXCR6 interacting with CXCL16, secreted by LSECs and KCs, and are activated upon engagement of the glycolipid receptor CD1d. Last, CD19^+^ B cells exert their functions through antibody production, antigen presentation and immune cell regulation.

Liver injury, caused by viral infection or chronic steatohepatitis related to alcohol or metabolic disorders, triggers an inflammatory cell death, leading to DAMP release and the influx of immune cells. Chronic inflammation activates HSCs, the main actors in liver fibrosis that produce extracellular matrix (ECM) components, forming the so-called “scar tissue.” Liver cirrhosis, which affects 1% of the world population, represents the soil where most HCC cases develop. Indeed, continuous cellular stress, repetitive cycles of necrosis and compensatory regeneration of parenchymal cells and chronic inflammation elicit cellular senescence and mutagenesis leading eventually to HCC development. Furthermore, a reduction of sinusoid porosity (defenestration), associated with collagenization of the space of Disse, was shown to impede immunosurveillance [reviewed in (23)].

**Figure 3:**
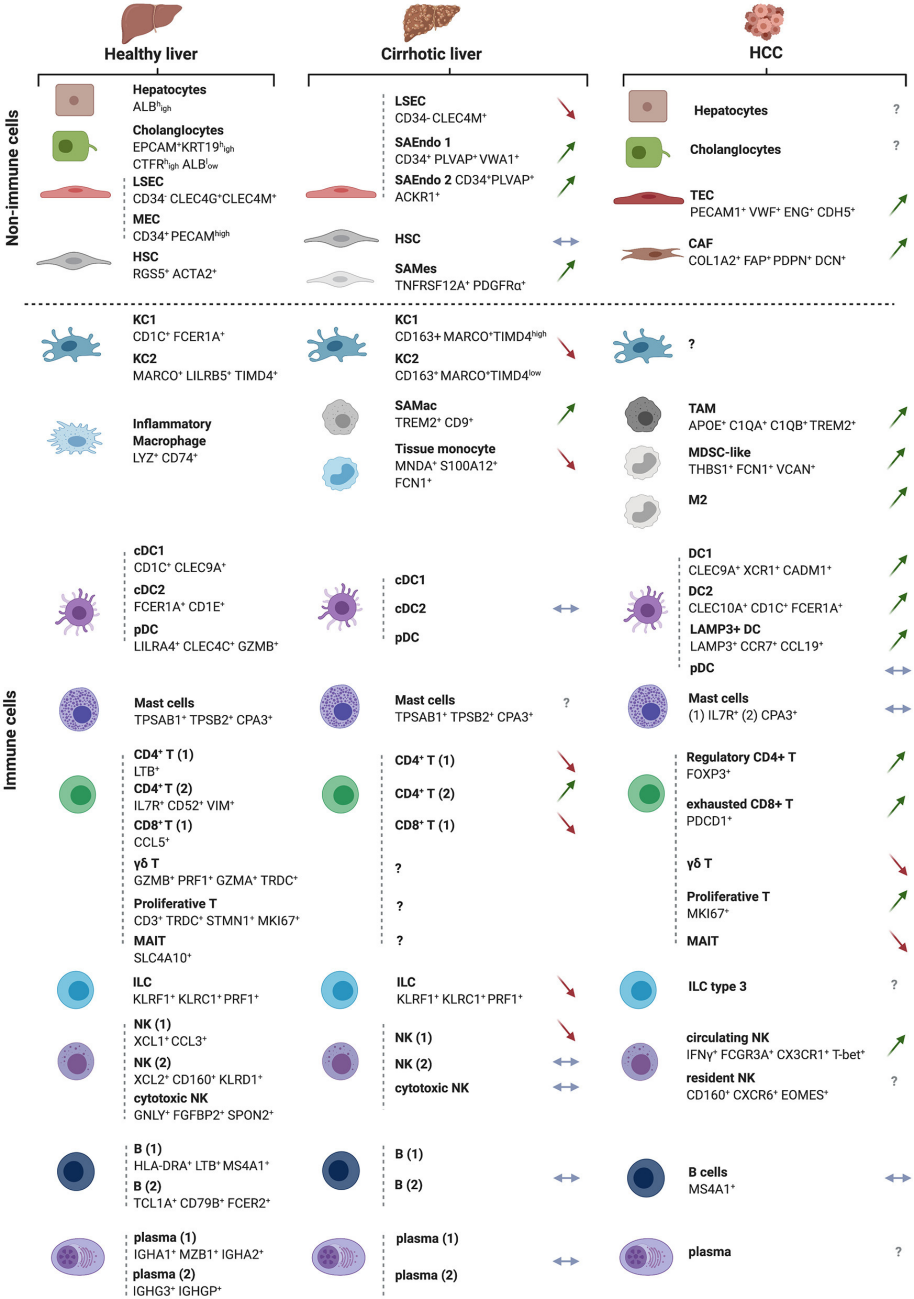
The landscapes of the normal, cirrhotic and HCC-bearing livers. All immune and non-immune cell types identified by high-resolution single cell analyses of the human healthy and cirrhotic livers and of HCC are illustrated along with their discriminatory markers. Arrows depict direction of change in cirrhosis or HCC vs. the normal liver, with green arrows indicating an expansion, red arrows a depletion and blue horizontal arrow no change in the examined cell subset. The cellular landscapes of the healthy and cirrhotic livers were from (26). The information on the HCC landscape was from (27), but with complementary information from the following studies: γδ T cells and M2 macrophages (28), cancer-associated fibroblasts (CAFs) and tumor-endothelial cells (TECs) (29). New subsets of cells arising in the cirrhotic condition are also depicted and labeled as ‘scar-associated’ cells: SAEndo: scar-associated endothelial cells; SAMes: scar-associated mesenchymal cells; SAMac: scar-associated macrophages, as in (26). The HCC analyses were on sorted CD45^+^ immune cells. Symbols for genes and associated proteins are defined in Supplementary Table 1.

The recent use of high-dimensional single cell approaches (e.g., mass cytometry and single cell RNA sequencing [scRNAseq]) in humans has unraveled the cellular landscape of the healthy (24, 25) and cirrhotic (26) livers and uncovered subtype heterogeneity for all major liver populations. According to two reports by Aizarani et al. (24) and MacParland et al. (25), in which parenchymal and non-parenchymal cells from dissociated human normal liver tissue were analyzed, the healthy liver is predominantly populated by leukocytes, which make up 45% of all liver cells, out-numbering hepatocytes (ALB^high^) that account for ~35% of the cells in this organ. This is followed by endothelial cells, including LSECs (CD34^−^ CLEC4G^+^ CLEC4M^+^) and macrovascular endothelial cells (CD34^+^ PECAM^high^) that account for ~7.5% and ~2.5% of hepatic cells, respectively. HSCs (RGS5^+^ ACTA2^+^) are found at <1% of the cells in this organ whereas cholangiocytes (EPCAM^+^ KRT19^high^ CTFR^high^ ALB^low^) occupy ~9% of the liver cellular landscape (24, 25) (Figure 3). Interestingly, among the EpCAM^+^ cholangiocytes, a putative bipotent liver progenitor population was identified by Aizarani et al. (24) based on the expression of intermediate levels of the intracellular calcium signal transducer *TACTSD2/TROP2* (TROP2^int^). This population was shown to give rise to ASGR1^+^ hepatocyte-biased cells (TROP2^low^) or KRT19^high^ CFTR^high^ ALB^low^ cholangiocytes (TROP2^hi^) (24). Furthermore, to model liver zonation, Aizarani et al. (24) applied diffusion pseudotime analysis and showed that hepatocytes and LSECs gene expression is highly zonated. LSECs in the periportal zone expressed genes involved in hormone signaling and metabolism, whereas pericentral and mid zone LSECs and hepatocytes were enriched in gene expression related to platelet activation, immune regulation and scavenging.

Among the leukocytes, the ratio of lymphocytes to mononuclear phagocytes (MNPs) is 3:1, with the former occupying ~35% and the latter 10% of total liver cells. The lymphocytic compartment includes ~11% αβ T cells, ~6.7% γδ T cells, ~12.3% NK + NKT cells, and ~ 5% B cells (25). Among the innate immune cells, NK cells cluster in three groups, NK1 (XCL1^+^ CCL3^+^), NK2 (XCL2^+^ CD160^+^ KLRD1^+^) and cytotoxic NKs (GNLY^+^ FGFBP2^+^ SPON2^+^), whereas MNPs consist of three subsets, including two CD68^+^ KC clusters, KC1 (CD1C^+^ FCER1A^+^) and KC2 (MARCO^+^ LILRB5^+^TIMD4^+^) and a liver resident inflammatory macrophage subset (LYZ^+^ CD74^+^). The 3 classical dendritic cell (DC) subsets were also identified, namely conventional DCs, cDC1 (CD1C^+^ CLEC9A^+^) and cDC2 (FCER1A^+^ CD1E^+^), and plasmacytoid DCs (LILRA4^+^ CLEC4C^+^ GZMB^+^) (Figure 3).

In the cirrhotic liver, scRNAseq uncovered all major immune cell populations and revealed a decrease in CD8^+^ T cells, associated with an increase in CD4^+^ T cells, as compared to the healthy liver. Re-clustering of MNPs identified four subgroups, annotated as KC1 (CD163^+^ MARCO^+^ TIMD4^hi^), KC2 (CD163^+^ MARCO^+^ TIMD4^low^), scar-associated macrophages (TREM2^+^ CD9^+^), tissue monocytes (MNDA^+^ S100A12^+^ FCN1^+^) (Figure 3). The MARCO^+^ population decreases in cirrhosis compared to the healthy liver while TREM2^+^ CD9^+^ scar-associated macrophages, derived from circulating monocytes, expand early in the course of the disease. This latter population of cells is conserved in humans and mice and displays pro-fibrogenic properties (26). Deep clustering of mesenchymal cell populations uncovered a cluster of PDGFα^+^ cells that also expand in cirrhosis, expressing high levels of fibrillar collagens and pro-fibrogenic genes. RNA velocity experiments indicated a trajectory from human HSCs to these scar-associated mesenchymal cells, and ligand/cognate receptors analysis combined with functional studies, pointed to TNFRSF12A, PDGFRA, and Notch signaling as important regulators of mesenchymal cell function in the human liver fibrotic niche (26).

Collectively, these single cell analyses revealed context-dependent cellular phenotypic diversity, opening the field to exploring potential mechanisms involved in HCC progression from cirrhosis. For instance, the fibrotic context is associated with the emergence of scar-associated mesenchymal cells and scar-associated macrophages with pro-fibrogenic properties. Future functional studies are needed to determine the value of targeting these cell subsets or specific molecular effectors therein as therapeutic strategies in HCC.

## HCC Subtypes According to Molecular and Immune Classifications

### Molecular Classification of HCC

**Figure 4:**
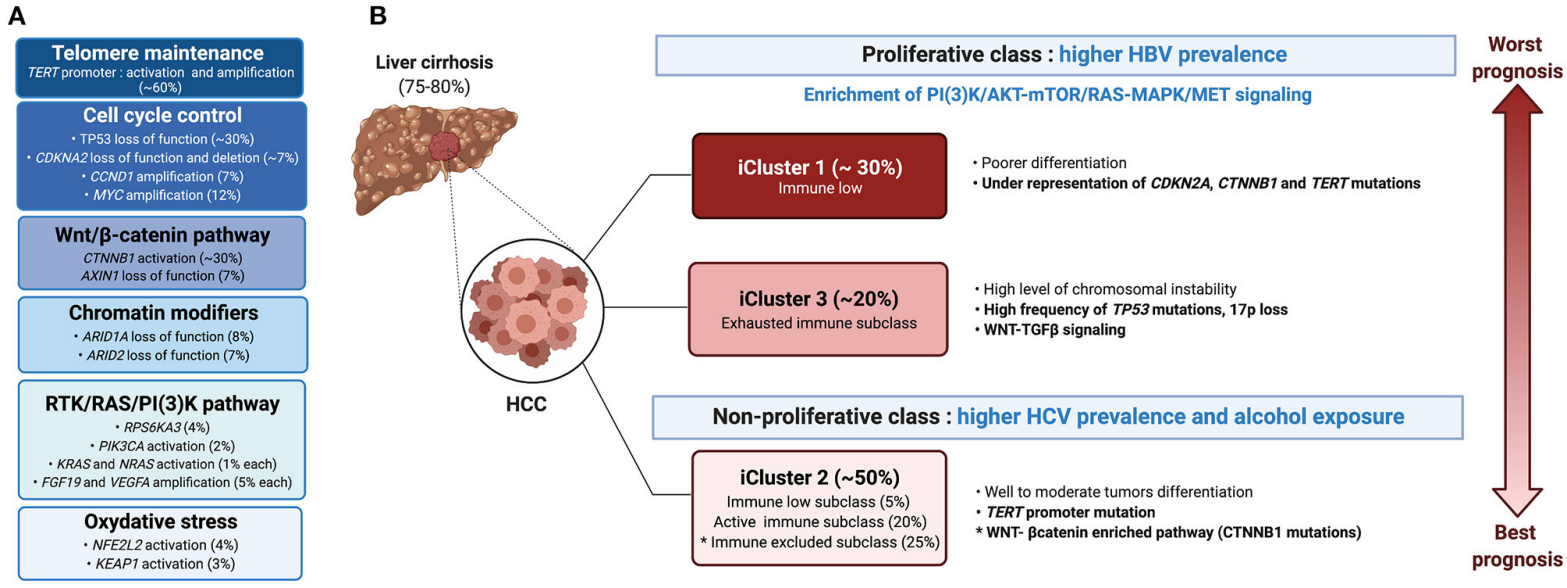
Molecular and immunological classifications of HCC. **(A)** The main oncogenic events associated with HCC are presented in descending order of incidence, the most common being defects in telomere maintenance, followed by alterations in cell cycle control, and activation of the WNT/β-catenin pathway. **(B)** Based on molecular features, HCC patients can be grouped into either the proliferative or non-proliferative class, the first with higher prevalence of HBV infection and a bad prognosis, whereas the second including cases with HCV infection or alcohol abuse and having a better prognosis. Integration of genomic, expression and epigenetic data by the TCGA research network (32) identified a different classification, namely iClusters 1-3. iCluster 2 which represents 50% of the patients and is related to the non-proliferative class, includes three sub-classes: an “active immune subclass,” an “immune excluded” subclass and an “immune low” subclass. An “immune exhausted” subclass is found within iCluster 3 of the proliferative class, whereas iCluster 1 is characterized by an “immune low” signature.

Progression from cirrhosis to HCC is mediated by a step-wise accumulation of somatic mutations and copy number variations in driver genes (30). The most frequent alteration is the reactivation of the telomerase reverse transcriptase (*TERT*), a key event observed in 20% of high-grade dysplastic lesions and up to 60% of early HCC (31). Besides *TERT* promoter mutations that impact telomere maintenance, 10 pathways were found to be recurrently altered in HCC, including pathways involved in cell cycle control (*TP53, CDKNA2, CCND1*), oxidative stress (*NFE2L2, KEAP1*), and chromatin modification (*ARID1A, ARID2*), but also the Wnt/β-catenin pathway (*CTNNB1, AXIN1*) and the RTK/RAS/PI3K pathway (*RPS6KA3, PIK3CA, KRAS, NRAS, FGF19, VEGFA*) (32) (Figure 4). The TGFβ pathway is additionally involved in HCC progression, with some tumors presenting aberrant activation of this pathway, whereas others harboring inactivating mutations in genes required for TGFβ signal transduction e.g., the *SPTBN1* gene (33). Last, ~20% of HCC express markers of progenitor cells, e.g., epithelial cell adhesion molecule (EpCAM) and cytokeratin 19 (CK19) and arise from either progenitors or dedifferentiated hepatocytes (12).

Earlier studies classified HCC into two main transcriptome-based classes, based on genetic, epigenetic and phenotypic features: HCC of the proliferative class, which displayed a poor clinical outcome, and HCC of the non-proliferative class, with a better outcome. The proliferative class was associated with the HBV etiology, and characterized by the activation of PI3K–AKT–mTOR, RAS–MAPK, and MET signaling along with chromosomal instability (34). The non-proliferative class, which is more prevalent in alcohol- or HCV-related HCC, regrouped heterogeneous tumors, including a subclass characterized by mutations in *CTNNB1*, the gene encoding β-catenin. More recent classification by Schulze et al. (35) and the Cancer Genome Atlas (TCGA) network (32) revised the molecular landscape of HCC (Figure 4). Three integrative clusters were identified: whereas, iCluster 1 and iCluster 3 distinguished two subclasses of the proliferative class, iCluster 2 overlapped with the non-proliferative class. iCluster 1 is associated with poorer differentiation, higher tumor grade, the presence of macrovascular invasion and overexpression of proliferation (*PLK1, MKI67*) and progenitor cells (EPCAM and AFP) gene markers, while iCluster 3 is characterized by high frequency of *TP53* mutation, 17p loss and activation of WNT-TGFβ signaling. On the other end, iCluster 2 regroups heterogenous moderately differentiated tumors characterized by *TERT* promotor mutations. Identification of the mutational landscape of HCC unveiled several druggable targets in >25% of the cases (35). However, a potential limitation of tumor cell-based therapy is a notable inter- and intra-tumoral heterogeneity, mediated in part by non-neutral selection of mutations conferring a selective advantage (30) and subclone evolution (36). Using scRNAseq of liver cancers, Ma et al. (29) identified links between intra-tumoral heterogeneity (ITH), tumor micro-environment (TME) and survival outcome. They discriminated ITH according to the average expression of 10 cancer stemness genes, namely *EPCAM, CD24, CD44, CD47, KRT19, PROM1, ALDH1A1, ANPEP, ICAM1* and *SOX9*. This allowed them to derive diversity scores based on transcriptomic profiles, grouping the tumors into Div-high and Div-low groups. The Div-high group displayed poorer mOS and PFS, expressed higher levels of hypoxia-inducible factor 1-alpha (HIF1α)-dependent VEGFA and displayed a marked TME reprogramming. Concordantly, NOTCH and VEGF signaling, together with fetal-associated endothelial cells (PLVAP^+^ VEGFR2^+^) found in tumors, have been demonstrated to reprogram the CD14^+^ monocytes into fetal-like immunosuppressive tumor-associated macrophages (TAMs (FOLR2^high^ CD163^high^) (37).

### Immunological Classification of HCC

Immunological classification of HCC has been proposed by different groups using gene expression profiling (38) and protein level approaches based on multiplex immunohistochemistry (IHC) analysis (38) and mass cytometry (CyTOF) (28). Using deconvolution of 8 datasets, Llovet and colleagues analyzed a total of 956 HCC samples and reported that ~25% of HCC cases expressed an immune gene signature (39). Such an “immune class” was found to be associated with better mOS, and expressed PD-1 and PD-L1, tertiary lymphoid structures (TLS) markers and determinants of cytolytic T cells activity e.g., an IFNγ signature. Further stratification identified two TME-based sub-classes within the immune class, dubbed the “active immune” and the “exhausted immune” subclasses. The “active immune” sub-class was enriched in T cell response effectors (IFNγ and granzyme B signatures), whereas the “exhausted immune” sub-class included signatures of T cell exhaustion, immunosuppressive macrophages and TGFβ signaling. A third immunological class, referred to as “immune excluded” was distinguished in ~25% of HCC patients, based on the expression of immune genes, particularly an immunosuppressive signature, in the tissue surrounding the tumor, but with little immune gene expression in the tumor core. Such an “immune excluded” class was associated with a bad prognosis and overlapped with a subset of tumors in TCGA iCluster 2 with an activated WNT-β-catenin pathway (39) (Figure 4B). The immunological environment of HCC and its association with the molecular classification was further analyzed by Kurebayashi et al. (38) using multiplex immunohistochemistry. The authors classified HCC into three immune-subtypes based on the numbers of infiltrating immune cells: “Immune-high,” “immune-mid” and “immune-low.” Consistent with Sia et al. (39), the “immune-high” subtype, which was enriched in T cells and B-/plasma cells, was associated with a good prognosis (38). Zhang et al. (28) expanded this analysis and defined three HCC groups, namely the “immunocompetent,” “immunosuppressive,” and “immunodeficient” subtypes. The immunocompetent subtype, characterized as CD45^high^ FOXP3^low^, had normal T cell infiltration including high infiltration of γδ T cells. On the contrary, the immunosuppressive subtype, marked by a CD45^high^ FOXP3^high^ staining, exhibited high frequencies of immunosuppressive cells (regulatory T and B cells and immunosuppressive macrophages) and molecules (PD-1, PD-L1, TIM-3, CTLA-4, VEGF, TGFβ, and IL-10). Finally, the CD45^low^ immunodeficient subtype showed a reduced infiltration of lymphocytes (28). While these studies demonstrated marked heterogeneity in HCC tumors and their associated TME with broad classification of patients, in depth characterization of the immune landscape of HCC at high resolution is expected to refine patients stratification and identify putative immune-therapeutic targets.

### The Immune Landscape of HCC

**Figure 5:**
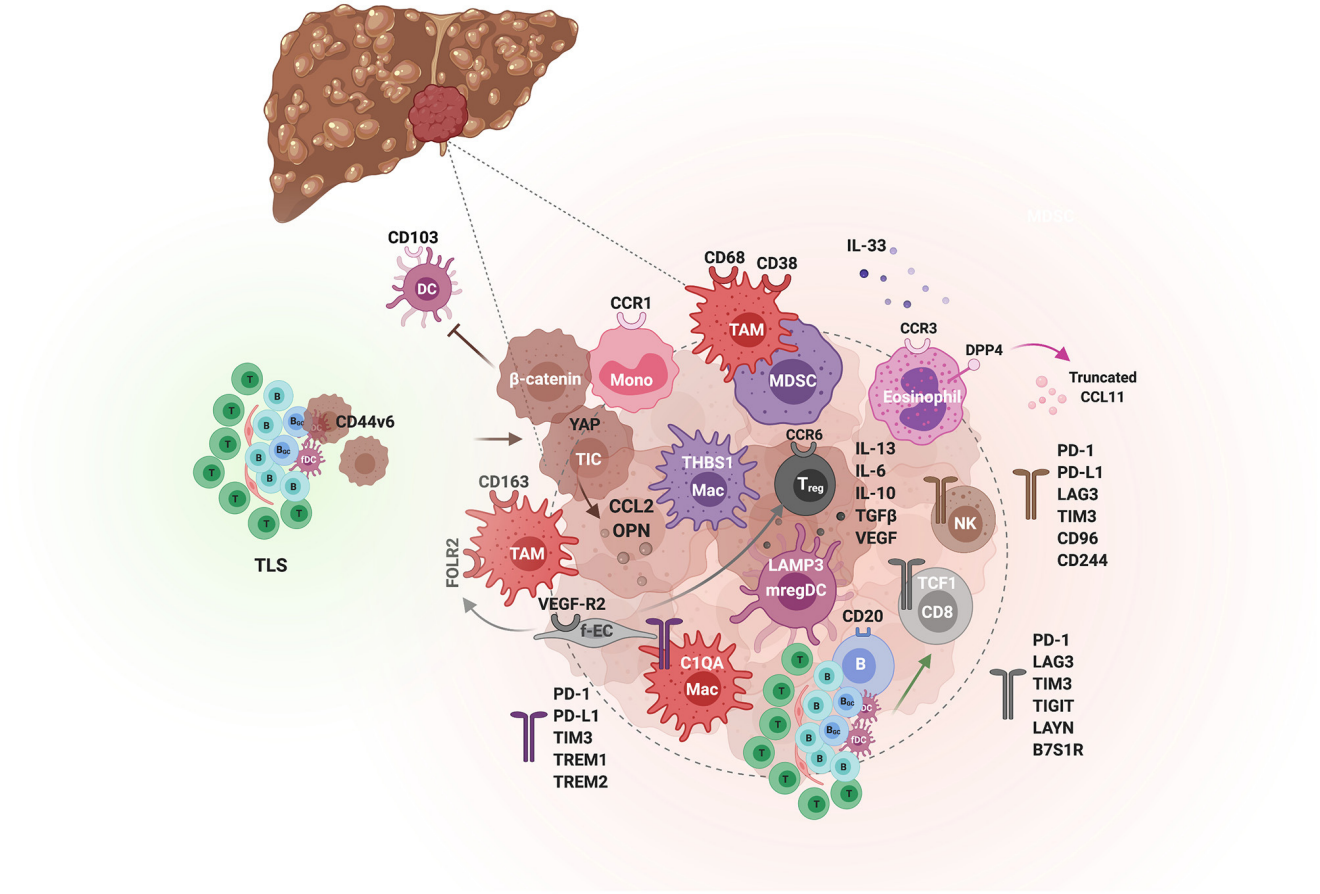
The tumor immunological microenvironment of HCC. Schematic illustration of the different actors demonstrated to contribute to immunosuppression or immune activity in the TME of HCC. Tumor-initiating cells (TIC) and tumor cells (hues of brown) orchestrate the immunological environment by secreting inflammatory cytokines e.g., osteopontin (OPN) and chemokines e.g., the monocyte chemoattractant CCL2, which promote tumorigenesis through the recruitment of monocytes and their differentiation to tumor-associated macrophages (TAMs). In addition, β-catenin-activated tumor cells can inhibit anti-tumor immunity by blocking CD103^+^ DCs tumor infiltration. Spatially, TAMs are enriched in the peri-tumoral area, express CD68, CD163, CD38, and the folate receptor (FOLR2), induced by fetal-associated endothelial cells (fEC). Two subsets of tumor-enriched macrophages can be distinguished by single cell analyses, THBS1^+^ macrophages and C1QA^+^ macrophages, with an MDSC and TAM signature, respectively. The latter is associated with a poor prognosis in HCC. The immunosuppressive activity of TAMs is mediated by various immune checkpoints, including PD-1, PD-L1, TIM-3, TREM1 and TREM2. Besides TAMs, the TME is enriched in Treg but depleted of other T cells and NK cells, which when present exhibit an exhausted phenotype. Among the CD8 T cells, The PD-1^+^ TCF1^+^ “precursors” are associated with a good response to anti-PD-1. TLS found in the adjacent non-tumoral liver tissue are associated with a poor prognosis as they can harbor progenitor/cancer stem cells (expressing CD44v6) and promote tumorigenesis by impairing the clearance of senescent hepatocytes. In contrast, intratumoral TLS and CD20^+^ B cells are predictive of a lower risk of relapse and a better CD8 T cell anti-tumor activity. Eosinophils also confer a tumorilytic activity, and enhancing their recruitment with inhibitors of the CCL11 peptidase DPP4 improved the efficacy of anti-PD-1+anti-CTLA4 immunotherapy.

The immune landscape of HCC has been more recently explored using single cell approaches. In general, a progressive depletion of intrahepatic LrNK cells, cytolytic T cells and γδ T cells and an enrichment of regulatory T cells (T_reg_) and macrophages occur in HCC (28, 28, 32, 40–42) (Figures 3, 5). While tumor-infiltrating CD8^+^ T cells are significantly correlated with better prognosis (38, 43), T_reg_ are associated with a poorer mOS (44). RNA velocity analysis indicated a directional flow from proliferative to exhausted CD8^+^ T cells in HCC (27). Exhaustion is characterized by the expression of a range of inhibitory receptors, including PD-1, TIM-3, LAG3, TIGIT, and LAYN [reviewed in (45)], and with reduced effector functions via TOX-mediated epigenetic and transcriptional alterations (46–48). However, not all exhausted CD8^+^ T cells are the same, as two subsets can be discriminated: PD-1^+^ TCF1^+^ “precursors” that self-renew and give rise to PD-1^+^ TCF1^−^ “terminally differentiated” exhausted T cells (49–51). Notably, the presence of the precursors, but not the terminally differentiated exhausted T cells, is associated with a better response to anti-PD-1. Similarly, NK cells display an exhausted phenotype, expressing high levels of immune checkpoints such as PD-L1, PD-1, LAG3, TIM-3, CD155, and CD96 (52, 53). Further, they produce immunosuppressive cytokines such as TGFβ and IL-10 and less IFN-γ (52–56). The role of B lymphocytes in the development of HCC and their prognostic value is still debated. Their ADCC and antigen-presentation functions are countered by their ability to induce immunosuppression. In surgically resected HCC, CD20^+^ B cells are associated with a better prognosis (38, 57), especially when they are in close proximity of tumor-infiltrating T cells (58). However, their prognostic value in the context of TLS depends on whether these are found intra-tumorally or in the surrounding tissue [reviewed in (59)]. Notably, TLS presence in the adjacent non-tumoral liver tissue was associated with an increased risk for late recurrence and a poor mOS in 82 patients with surgically resected HCC (60). Mechanistically, such ectopic TLS harbored progenitors/cancer stem cells (expressing CD44v6) and a tumor-promoting environment characterized by a persistent NF-κB activation favoring tumor outgrowth, as demonstrated in a mouse model (60). Concordantly, alymphoid conditions suppressed CD44v6^+^ HCC-initiating cells and prevented hepatocarcinogenesis (60, 61). Additionally, B cells favored HCC progression by limiting the resolution of senescence-mediated liver fibrosis (62). In contrast, it was recently shown that intra-tumoral TLS were predictive of a lower risk of early relapse after surgical resections, as analyzed in 273 patients (63). In contrast, CD68^+^ CD163^+^ TAMs, which accumulate at the tumor margin, and CCR1^+^ monocytes, are associated with bad mOS (64–66). TAMs are recruited to HCC in response to CCL2. The expression of the *CCL2* gene is controlled by diverse mechanisms including through APOBEC3B-mediated de-repression of epigenetic marks in its promoter (67), and through Yes-associated protein (YAP) transcriptional regulation, as shown in tumor-initiating cells (TICs) (68). Together with IL-13, CCL2 drives the metastasis of MYC/Twist1 tumors (69). TAMs contribute to HCC malignant progression and metastasis through diverse mechanisms, e.g., *via* the production of cytokines such as IL-6 (70) and hepatocyte compensatory proliferation (71), immunosuppression and induction of epithelial-to-mesenchymal transition (EMT) [reviewed in (72)]. The immune checkpoint TIM-3, induced by TGFβ in the tumor microenvironment (TME), is implicated in the pro-tumoral effects of TAMs in HCC (70). Similarly, the receptors Triggering Receptor Expressed on Myeloid cells (TREM)1 (73) and TREM2 (74) have been demonstrated to promote the dysfunction and apoptosis of cytotoxic CD8^+^ T cells in HCC, while enhancing the recruitment of CCR6^+^ Foxp3^+^ Tregs. Platelets, key effectors of immune-mediated tissue damage, have also been implicated in HCC. Using different mouse models of dietary-inducing NASH and data from human patients, Malehmir et al. (75) demonstrated enhanced platelets influx, aggregation and activation in liver sinusoids in NASH. This was mediated by their interaction with KCs, involving hyaluronic acid/CD44 binding and the platelet receptor glycoprotein 1b alpha (GPIbα). Anti-platelet treatments, including aspirin, or specific blockade of GPIbα blunted the development of NASH, through limiting CD8 T lymphocytes, NKT cells and KC recruitment. In the HBsAg transgenic mouse model of HCC, platelets were similarly shown to promote the recruitment of HBsAg-specific CD8 T cells that elicit cycles of hepatocyte killing and inflammation leading to fibrosis. Inhibition of platelet activation potently reduced the development of HCC in this model (76).

scRNAseq of sorted CD45^+^ cells from the tumor, adjacent liver, hepatic lymph nodes, blood, and ascites of 16 treatment-naive HCC patients recovered all of the major cell populations such as T, B, NK and myeloid cells, but also few minor cell populations including mast cells and ILCs (27) (Figure 3). All types of T cells (including Treg, exhausted T cells [Tex] and proliferative T cells) were enriched in the tumors, as previously reported (77). Four clusters of NK cells, enriched in the tumor, were identified, including two circulating NK clusters (IFNγ^+^ FCGR3A^+^ CX3CR1^+^ T-bet^+^) and two LrNK clusters (CD160^+^ CXCR6^+^ EOMES^+^). However, their respective roles in tumorigenesis and patients prognosis have not been addressed. A diverse repertoire of functionally distinct myeloid cells were identified, particularly, two subsets of macrophages within the tumors: THBS1+ macrophages enriched in myeloid-derived suppressor cell (MDSC) genes (S100A genes, FCN1 and VCAN) and C1QA+ macrophages, enriched in tumor associated macrophage (TAM) genes APOE, C1QB and TREM2 (Figures 3, 5). Only the latter was associated with a poor prognosis in the TCGA liver hepatocellular carcinoma (LIHC) cohort. In parallel, 3 intra-tumoral clusters of DCs were distinguished, namely cDC2 (highly expressing CD1C, FCER1A, and CLEC10A), cDC1 (highly expressing CLEC9A, XCR1 and CADM1) and a non-classical LAMP3^+^ DCs (highly expressing CCR7, LAMP3, CD80 and CCL19) with migration capacity toward the lymph nodes. Interestingly, ligand-receptor pairs analysis indicated that the LAMP3^+^ DCs are the subset that would interact with Tex cells and T_regs_. This LAMP3 population seems to correspond to mregDCs, a DC cluster annotated in human lung cancer as a population involved in tumor antigen uptake and expressing immunoregulatory molecules (78, 79).

### HCC Patients Response to ICIs

PD-1 is primarily expressed on the surface of activated T cells, but also on NK/NKT cells (54), B cells (80) and myeloid cells including monocytes, DCs, MDSCs and TAMs (81). Its induction in response to cytokine signaling is tightly regulated at the epigenetic and post-transcriptional levels (48, 81, 82). Recently, two studies used multiparametric flow cytometry and multiplex IHC to show that higher intratumoral frequency of PD-1^high^ CD8^+^ T cells (83) and CD38^+^ CD68^+^ macrophages (84) was strongly associated with improved response to ICI in patients with advanced HCC. PD-L1 is expressed by DCs, monocytes, macrophages, B cells, NK cells, LSECs, and tumor cells. Its expression is induced by hypoxia (73), among other mechanisms. PD-L1 expression on tumor-infiltrating immune cells is associated with a better prognosis while the prognostic value of its expression on neoplastic cells is controversial (39, 43). Further, the response of patients with HCC to Nivolumab (anti-PD1) was not found to be associated with PD-L1 expression on tumor cells, implicating other PD-L1 expressing cells in this response (15). In murine models with transplantable HCC, PD-L1 expression on myeloid cells mediated the anti-PD-L1 response (85).

Previous studies in melanoma and non-small cell lung cancer (NSCLC) have attributed the response to ICIs to tumor mutational burden (86, 87), levels of neo-antigens (88) or tumor-specific antigens (89, 90), the presence of TLS [reviewed in (59)] or specific oncogenic pathways (91, 92). Nonetheless, the mechanisms involved in patients response to ICIs, particularly in HCC, remain for the most part unclear. For example, the mutational burden did not correlate with ICI response in HCC (93), and neither the mutational load nor the presence of neoantigens was associated with the immune class, which predicted a favorable response to ICI therapy (39). Instead, the activation of β-catenin was associated with resistance to ICI, as demonstrated in a mouse model (94) (Figure 4). Using a *MYC;p53*^−/−^ HCC mouse model, Ruiz de Galarreta et al. (94) demonstrated that β-catenin promoted immune escape by preventing the recruitment of CD103^+^ DCs, impairing antigen-specific T cells-mediated anti-tumor immunity. Accordingly, activating mutations in *CTNNB1* correlate with resistance to ICI monotherapy with either anti-PD-1 or anti-PD-L1, as shown in a prospective sequencing analysis of 27 evaluable advanced HCC patients, in which none of the 10 patients with WNT pathway alterations achieved clinical benefit, whereas around half of the non-WNT pathway–altered patients showed durable stable disease (93). Nevertheless, these results also show that 50% of ICI non-responders harbor mechanisms unrelated to β-catenin activation. Treating fibrosis using a TGFβ neutralizing antibody in the STAM™ mouse model fibrosis-associated HCC, triggered a redistribution of CD8^+^ lymphocytes into the tumors, which re-invigorated anti-tumor response (95). These results are consistent with those of Mariathasan et al. (96), who reported that TGFβ attenuated the response to PD-L1 blockade by restricting intra-tumoral T-cell infiltration. Since TGFβ alterations are found in a subset of HCC patients (33), agents that block this pathway should be tested in this group, highlighting the need for personalized medicine. Similarly, the immunosuppressive molecule VEGF was found to be enriched in a subset of HCC patients, particularly those with Div-high tumors, supporting the use of the anti-PD-L1 (Atezolizumab) + anti-VEGF (Bevacizumab) combination. However, the cellular subsets, putative signaling pathways, and associated biomarkers required for an effective patient's response to this new combination therapy require further exploration. The etiology of HCC might contribute to the heterogeneity in patients' response to immunotherapy. Indeed, Lim et al. (44) reported that the TME of HBV-related HCC is more immunosuppressive than that of non-viral HCC. Particularly, PD-1^high^ Tregs and PD-1^+^ CD8^+^ resident memory T cells were more prominent in HBV-related HCC, suggesting that PD-1 blockade might be a suited strategy for this etiology. In contrast, immunotherapies that target CD244^+^ NK cells and Tim-3^+^ CD8^+^ T cells, enriched in non-viral HCC, may be more effective in those patients (44).

### ICI Combination Therapies

Several pre-clinical studies and ongoing clinical trials (Table 1) are exploring the potential of combining different ICIs. For e.g., a phase III trial is currently testing the combination of durvalumab (anti-PD-L1) and tremelimumab (anti-CTLA-4) as a first line therapy (NCT03298451). The combinations of ICIs together with ablation (97), chemo-radioembolization or targeted therapies (TKi or anti-VEGF) in the adjuvant or neoadjuvant setting are also being explored (Table 1). For e.g., two trials are testing the combination of pembrolizumab + Lenvatinib (NCT 03006926) or of pembrolizumab + regorafenib (NCT03347292) for first line therapies. Radioembolization was reported to elicit an immune response, both locally and systemically, leading to enhanced infiltration of TIM-3^+^ tumor-infiltrating lymphocytes (TILs), NK, and NKT cells (98). It is thus plausible that an ICI targeting TIM-3 might enhance the clinical response of radioembolization or other interventions in HCC patients. A phase II trial is currently testing cobolimab, a TIM-3 binding antibody, in combination with anti-PD-1 on the response of patients with locally advanced or metastatic liver cancer (NCT03680508). Similarly, combining multiple strategies targeting inhibitory receptors (PD-1, TIM-3, LAG3, CTLA4, TREM1, TREM2) and/or their ligands (PD-L1, B7 superfamily member1 [B7S1]) have shown synergistic effects in restoring TILs anti-tumoral immune responses in pre-clinical studies (73, 74, 99–102) and enhancing NK cell infiltration and activity (52–56, 103). Additional strategies include the inhibition of TAM recruitment, their polarization to an immunosuppressive phenotype or their function in hampering anti-tumor immunity or promoting tumorigenesis. The pro-inflammatory protein osteopontin (OPN) produced by cancer cells has been implicated in cancer promotion and metastasis, through the stimulation of CSF1 signaling in TAMs. Blockade of the CSF1/CSF1R pathway enhanced the efficacy of anti PD-L1 in OPN-overexpressing HCC, by reducing macrophage recruitment (102). Blockade of the CCL2/CCR2 axis was also shown to inhibit the recruitment of TAMs leading to enhanced infiltration of CD8^+^ T cells and improved anti-tumor immunity (104). However, this approach should be considered with caution as some macrophages exert anti-tumoral activity. Indeed, Eggert et al. (105) reported that the CCL2-CCR2 axis promotes the clearance of senescent hepatocytes preventing HCC outgrowth in mice. Among the TAM targets that recently surfaced as critical inhibitors of anti-tumor immunity are the receptors TREM1 and TREM2. Blockade of TREM1 (73) or TREM2 (74) attenuated immunosuppression and CD8^+^ T cell dysfunction boosting the efficacy of anti-PD-1/PD-L1 immunotherapy. An alternative approach to skew TAM functions is through vaccination. Using a mouse model, a recent study demonstrated that a *Listeria*-based HCC vaccine enhanced the efficacy of PD-1 blockade by skewing the TAMs to an anti-tumoral phenotype (106). Consistent with the improved patients response to the anti-PD-L1 + anti-VEGF combination therapy, it has been recently demonstrated using murine models of HCC that this approach fortified hepatic vessels and overcame resistance to either monotherapy (107). Last, a less studied immune cell population in the context of anti-tumor immunity are the eosinophils, which were recently shown in a murine model of HCC to promote tumor-cell killing through degranulation and contribute to the efficacy of the anti-PD1 + anti-CTLA4 combination immunotherapy. Their recruitment in response to the cancer-cell secreted alarmin IL-33, is mediated by the chemokine CCL11, and enhanced with the administration of sitagliptin, an inhibitor of dipeptidyl peptidase DPP4 (CD26) that cleaves CCL11. These results suggest that combined modulation of both type 1 and 2 immune responses may improve therapeutic management of HCC (108).

**Table 1:** Clinical trials of immunotherapies for HCC.

**Immunotherapy**	**Identifier**	**Study title**	**Interventions**	**Number enrolled**	**Primary completion**
**Phase III clinical trials**					
***ICI + combinations***					
**ICI as Adjuvant (Stage A)**	NCT03867084	Safety and Efficacy of Pembrolizumab (MK-3475) vs. Placebo as AdjuvantTherapy in Participants With Hepatocellular Carcinoma(HCC) and CompleteRadiological Response After Surgical Resection or Local Ablation (MK-3475-937/KEYNOTE-937)	Biological: Pembrolizumab Drug: Placebo	950	June 2025
	NCT03383458	A Study of Nivolumab in Participants With Hepatocellular Carcinoma Who Are at High Risk of Recurrence After Curative Hepatic Resection or Ablation (CheckMate 9DX)	Biological: Nivolumab Other: Placebo	530	Jan 2023
	NCT04102098	A Study of Atezolizumab Plus Bevacizumab vs. Active Surveillance as Adjuvant Therapy in Patients With Hepatocellular Carcinoma at High Risk of Recurrence After Surgical Resection or Ablation (IMbrave050)	Drug: Atezolizumab Drug: Bevacizumab	662	Mar 2023
	NCT03847428	Assess Efficacy and Safety of Durvalumab Alone or Combined With Bevacizumab in High Risk of Recurrence HCC Patients After Curative Treatment (EMERALD-2)	Drug: Durvalumab Drug: Bevacizumab Other: Placebo	888	Sept 2022
	NCT03859128	Toripalimab or Placebo as Adjuvant Therapy in Hepatocellular Carcinoma After Curative Hepatic Resection (JUPITER 04)	Biological: TORIPALIMAB INJECTION (JS001)	402	Oct 2022
*ICI + TACE* *(Stage B)*	NCT04229355	DEB-TACE Plus Lenvatinib or Sorafenib or PD-1 Inhibitor for Unresectable Hepatocellular Carcinoma	Drug: DEB-TACE plus Sorafenib Drug: DEB-TACE plus Lenvatinib Drug: DEB-TACE plus PD-1 inhibitor	90	Dec 2022
	NCT04246177	Safety and Efficacy of Lenvatinib (E7080/MK-7902) With Pembrolizumab (MK-3475) in Combination With Transarterial Chemoembolization (TACE) in Participants With Incurable/Non-metastatic Hepatocellular Carcinoma (MK-7902-012/E7080-G000-318/LEAP-012)			
	NCT03949231	Infusion of Toripalimab Via Hepatic Arterial vs. Vein for Immunotherapy of Advanced Hepatocellular Carcinoma	Drug: Toripalimab	200	Jan 2022
	NCT03755739	Trans-Artery/Intra-Tumor Infusion of Checkpoint Inhibitors for Immunotherapy of AdvancedSolid Tumors	Drug: Checkpoint inhibitor (CPI) such as Pembrolizumab	200	Nov 2033
	NCT04268888	Nivolumab in Combination With TACE/TAE for Patients With Intermediate Stage HCC	Drug: Nivolumab and TACE/TAE Procedure: TACE/TAE	522	June 2025
	NCT0378957	A Global Study to Evaluate Transarterial Chemoembolization (TACE) in Combination With Durvalumab and Bevacizumab Therapy in Patients With Locoregional Hepatocellular Carcinoma (EMERALD-1)	Drug: Durvalumab Drug: Bevacizumab Other: Placebo Procedure: Transarterial Chemoembolization (TACE)	600	Aug 2021
*ICI + stereotaxic radiotherapy* *(Stage B)*	NCT04167293	Combination of Sintilimab and Stereotactic Body Radiotherapy in Hepatocellular Carcinoma (ISBRT01)	Radiation: stereotactic body radiotherapy Drug: Sintilimab	116	Nov 2021
*Monotherapy* *(Stage C)*	NCT02576509	An Investigational Immuno-therapy Study of Nivolumab Compared to Sorafenib as a First Treatment in Patients With Advanced Hepatocellular Carcinoma	Drug: Nivolumab Drug: Sorafenib	743	May 2019
	NCT03412773	Phase 3 Study of Tislelizumab vs. Sorafenib in Participants With Unresectable HCC	Drug: Tislelizumab Drug: Sorafenib	674	June 2021
*ICI +* α*VEGF (Stage C)*	NCT03434379	A Study of Atezolizumab in Combination With Bevacizumab Compared With Sorafenib in Patients With Untreated Locally Advanced or Metastatic Hepatocellular Carcinoma [IMbrave150] (IMbrave150)	Drug: Atezolizumab Drug: Bevacizumab Drug: Sorafenib	480	Feb 2021
	NCT03794440	A Study to Evaluate the Efficacy and Safety of Sintilimab in Combination With IBI305 (Anti-VEGF Monoclonal Antibody) Compared to Sorafenib as the First-Line Treatment for Advanced Hepatocellular Carcinoma.	Drug: Sintilimab Drug: IBI305 Drug: Sorafenib	566	Dec 2022
*ICI + TKi (Stage C)*	NCT03713593	Safety and Efficacy of Lenvatinib (E7080/MK-7902) in Combination With Pembrolizumab (MK-3475) vs. Lenvatinib as First-line Therapy in Participants With Advanced Hepatocellular Carcinoma (MK-7902-002/E7080-G000-311/LEAP-002)	Drug: lenvatinib Biological: pembrolizumab Drug: saline placebo	750	May 2022
	NCT03764293	A Study to Evaluate SHR-1210 in Combination With Apatinib as First-Line Therapy in Patients With Advanced HCC	Drug: SHR-1210 Drug: Apatinib Drug: Sorafenib	510	Dec 2021
	NCT03755791	Study of Cabozantinib in Combination With Atezolizumab vs. Sorafenib in Subjects With Advanced HCC Who Have Not Received Previous Systemic Anticancer Therapy (COSMIC-312)	Drug: Cabozantinib Drug: Sorafenib Drug: Atezolizumab	740	June 2021
*ICI + ICI (Stage C)*	NCT03298451	Study of Durvalumab and Tremelimumab as First-line Treatment in Patients With Advanced Hepatocellular Carcinoma (HIMALAYA)	Drug: Durvalumab Drug: Tremelimumab (Regimen 1) Drug: Tremelimumab (Regimen 2) Drug: Sorafenib Drug: Durvalumab (Regimen 1) Drug: Durvalumab (Regimen 2)	1324	Dec 2020
	NCT04039607	A Study of Nivolumab in Combination With Ipilimumab in Participants With Advanced Hepatocellular Carcinoma (CheckMate 9DW)	Drug: Nivolumab Drug: Ipilimumab Drug: Sorafenib Drug: lenvatinib	650	Mar 2023
***ACT***	NCT02678013	RFA+Highly-purified CTL vs. RFA Alone for Recurrent HCC	Procedure: RFA Procedure: RFA+highly-purified CTL	210	Jan 2020
	NCT02709070	Resection+Highly Purified CTL vs. Resection Alone for HCC	Procedure: resection Procedure: highly-purified CTL	210	Mar 2020
	NCT03592706	Autologous Immune Killer Cells to Treat Liver Cancer Patients as an Adjunct Therapy	Biological: IKC (Immune Killer Cells) Procedure: TACE (Transcatheter Arterial Chemoembolization)	60	Feb 2021
***OV***	NCT02562755	Hepatocellular Carcinoma Study Comparing Vaccinia Virus Based Immunotherapy PlusSorafenib vs. Sorafenib Alone	Biological: Pexastimogene Devacirepvec (Pexa Vec) Drug: Sorafenib	600	Dec 2020
***Vax***	NCT02232490	Liver Cancer Immunotherapy: Placebo-controlled Clinical Trial of Hepcortespenlisimut-L	Biological: hepcortespenlisimut-L Biological: Placebo	120	Nov 2019
**Phase II clinical trials**					
***ICI + combinations***					
Neoadjuvant (Stage A)	NCT03222076	Nivolumab With or Without Ipilimumab in Treating Patients With Resectable Liver Cancer	Biological: Ipilimumab Biological: Nivolumab	30	Sept 2022
	NCT03510871	Nivolumab Plus Ipilimumab as Neoadjuvant Therapy for Hepatocellular Carcinoma (HCC)	Drug: nivolumab, ipilimumab	40	Dec 2022
	NCT03630640	Neoadjuvant and Adjuvant Nivolumab in HCC Patients Treated by Electroporation	Drug: Nivolumab Injection [Opdivo]	50	Sept 2020
	NCT03682276	Safety and Bioactivity of Ipilimumab and Nivolumab Combination Prior to Liver Resection in Hepatocellular Carcinoma	Biological: Ipilimumab Biological: Nivolumab	32	Dec 2020
	NCT04174781	Neoadjuvant Therapy for Hepatocellular Carcinoma	Drug: Sintilimab Injection Drug: TACE	61	Nov 2020
*ICI + TACE (Stage B)*	NCT03638141	CTLA-4 /PD-L1 Blockade Following Transarterial Chemoembolization (DEB-TACE)in Patients With Intermediate Stage of HCC (Hepatocellular Carcinoma) Using Durvalumab and Tremelimumab	Drug: Durvalumab Drug: Tremelimumab (Cohort A dose) Drug: Tremelimumab (Cohort B dose)	30	Nov 2020
	NCT04273100	PD-1 Monoclonal Antibody, Lenvatinib and TACE in the Treatment of HCC	Combination Product: PD-1 mAb combined with TACE and lenvatinib	56	Dec 2020
	NCT03817736	Sequential TransArterial Chemoembolization and Stereotactic RadioTherapy WithImmunoTherapy for Downstaging Hepatocellular Carcinoma for Hepatectomy	Procedure: TACE Radiation: SBRT Drug: Immune Checkpoint Inhibitor	33	Feb 2022
	NCT04522544	Durvalumab (MEDI4736) and Tremelimumab in Combination With Either Y-90 SIRT orTACE for Intermediate Stage HCC With Pick-the-winner Design	Drug: Tremelimumab Drug: Durvalumab Procedure: Y-90 SIRT Procedure: TACE	84	Mar 2024
	NCT04518852	TACE, Sorafenib and PD-1 Monoclonal Antibody in the Treatment of HCC	Combination Product: TACE combined with sorafenib and PD-1 mAb	60	July 2022
	NCT03937830	Combined Treatment of Durvalumab, Bevacizumab, Tremelimumab and TransarterialChemoembolization (TACE) in Subjects With Hepatocellular Carcinoma or Biliary Tract Carcinoma	Drug: durvalumab Drug: Doxorubicin-Eluting Beads Procedure: TACE (and 2 more…)	22	Dec 2022
	NCT03817736	Sequential TransArterial Chemoembolization and Stereotactic RadioTherapy With ImmunoTherapy for Downstaging Hepatocellular Carcinoma for Hepatectomy	Procedure: TACE Radiation: SBRT Drug: Immune Checkpoint Inhibitor	33	Feb 2022
	NCT04268888	Nivolumab in Combination With TACE/TAE for Patients With Intermediate Stage HCC	Drug: Nivolumab and TACE/TAE Procedure: TACE/TAE	522	June 2025
	NCT03259867	Combination of TATE and PD-1 Inhibitor in Liver Cancer	Drug: Opdivo Injectable Product or Keytruda Injectable Product Combination Product: Trans-arterial tirapazamine embolization	80	Oct 2020
	NCT04191889	A Trial of Hepatic Arterial Infusion Combined With Apatinib and Camrelizumab for C-staged Hepatocellular Carcinoma in BCLC Classification	Combination Product: Hepatic Arterial Infusion combined with Apatinib and Camrelizumab	84	Dec 2021
	NCT03397654	Study of Pembrolizumab Following TACE in Primary Liver Carcinoma (PETAL)	Drug: Pembrolizumab Combination Product: Trans-arterial chemoembolization	26	Mar 2020
*ICI + radioembolization Thermal ablation and radiotherapy (Stage B)*	NCT03033446	Study of Y90-Radioembolization With Nivolumab in Asians With Hepatocellular Carcinoma	Radiation: Y-90 Radioembolization Drug: Nivolumab	40	Dec 2019
	NCT03380130	A Study of the Safety and Antitumoral Efficacy of Nivolumab After SIRT for the Treatment of Patients With HCC (NASIR-HCC)	Drug: Nivolumab Device: SIR-Spheres	40	Oct 2019
	NCT03753659	IMMULAB - Immunotherapy With Pembrolizumab in Combination With Local Ablation in Hepatocellular Carcinoma (HCC)	Drug: Pembrolizumab Procedure: Radio Frequency Ablation (RFA) Procedure: Microwave Ablation (MWA) (and 2 more…)	30	Mar 2022
	NCT04193696	RT+ Anti-PD-1 for Patients With Advanced HCC (RT+PD-1-HCC)	Drug: Radiation therapy and systemic anti-PD-1 immunotherapy for patients with advanced hepatocellular carcinoma	39	June 2020
	NCT03864211	Thermal Ablation Followed by Immunotherapy for HCC	Procedure: Thermal ablation Drug: Toriplimab	120	Mar 2021
	NCT04167293	Combination of Sintilimab and Stereotactic Body Radiotherapy in Hepatocellular Carcinoma (ISBRT01)	Radiation: stereotactic body radiotherapy Drug: Sintilimab	116	Nov 2021
	NCT03316872	Study of Pembrolizumab and Radiotherapy in Liver Cancer	Drug: Pembrolizumab Radiation: Stereotactic Body Radiotherapy (SBRT)	30	Apr 2020
*Monotherapy Stage C)*	NCT01693562	A Phase 1/2 Study to Evaluate MEDI4736	Drug: MEDI4736	1022	Feb 2020
	NCT03389126	Phase II Study of Avelumab in Patients With Advanced Hepatocellular Carcinoma After Prior Sorafenib Treatment (Avelumab HCC)	Drug: Avelumab	30	Dec 2019
ICI + TKi (Stage C)	NCT01658878	An Immuno-therapy Study to Evaluate the Effectiveness, Safety and Tolerability of Nivolumab or Nivolumab in Combination With Other Agents in Patients With Advanced Liver Cancer	Biological: Nivolumab Drug: Sorafenib Drug: Ipilimumab Drug: Cabozantinib	1097	Aug 2020
	NCT03841201	Immunotherapy With Nivolumab in Combination With Lenvatinib for Advanced Stage Hepatocellular Carcinoma	Drug: Lenvatinib Drug: Nivolumab	50	July 2021
	NCT04183088	Regorafenib Plus Tislelizumab as First-line Systemic Therapy for Patients With Advanced Hepatocellular Carcinoma	Drug: Tislelizumab+ regorafenib for part 1;Tislelizumab+ regorafenib for group 1 of part 2; Placebo+regorafenib for group 2 of part 2.	125	Mar 2024
	NCT04310709	Combination of Regorafenib and Nivolumab in Unresectable Hepatocellular Carcinoma	Drug: Regorafenib/Nivolumab	42	May 2022
	NCT03439891	Sorafenib and Nivolumab in Treating Participants With Unresectable, Locally Advanced or Metastatic Liver Cancer	Other: Laboratory Biomarker Analysis Biological: Nivolumab Drug: Sorafenib	40	Sept 2022
	NCT03170960	Study of Cabozantinib in Combination With Atezolizumab to Subjects With LocallyAdvanced or Metastatic Solid Tumors	Drug: cabozantinib Drug: atezolizumab	1732	Dec 2020
	NCT03899428	Immune Checkpoint Therapy vs. Target Therapy in Reducing Serum HBsAg Levels inPatients With HBsAg+ Advanced Stage HCC	Drug: Durvalumab Drug: Sorafenib Drug: Lenvatinib (and 2 more…)	30	Dec 2021
	NCT04442581	Cabozantinib and Pembrolizumab for the First-Line Treatment of Advanced Liver Cancer	Drug: Cabozantinib S-malate Biological: Pembrolizumab	29	Sept 2023
	NCT04523662	Study on the Effectiveness and Safety of Carrelizumab Combined With Apatinib Mesylate and Radiotherapy in the Treatment of Advanced Liver Cancer	Drug: Camrelizumab Apatinib Mesylas	27	Aug 2022
	NCT04212221	MGD013 Monotherapy and Combination With Brivanib Dose Escalation and Expansion Study in Advanced Liver Cancer Patients	Drug: MGD013 monotherapy Drug: MGD013 in combination with Brivanib Alaninate	300	Dec 2022
	NCT03463876	A Trial of SHR-1210 (an Anti-PD-1 Inhibitor) in Combination With Apatinib in Patients With Advanced HCC(RESCUE)	Drug: SHR 1210+apatinib	190	June 2019
*ICI + ICI*	NCT03228667	QUILT-3.055: A Study of Combination Immunotherapies in Patients Who Have Previously Received Treatment With Immune Checkpoint Inhibitors	Drug: N-803 + Pembrolizumab Drug: N-803 + Nivolumab Drug: N-803 + Atezolizumab (and 7 more…)	636	June 2021
	NCT03311334	A Study of DSP-7888 Dosing Emulsion in Combination With Immune CheckpointInhibitors in Adult Patients With Advanced Solid Tumors	Drug: DSP-7888 Dosing Emulsion Drug: Nivolumab Drug: Pembrolizumab	84	Nov 2021
	NCT03228667	QUILT-3.055: A Study of Combination Immunotherapies in Patients Who Have PreviouslyReceived Treatment With Immune Checkpoint Inhibitors	Drug: N-803 + Pembrolizumab Drug: N-803 + Nivolumab Drug: N-803 + Atezolizumab (and 7 more…)	636	June 2021
	NCT04430452	Hypofractionated Radiotherapy Followed by Durvalumab With or Without Tremelimumab for the Treatment of Liver Cancer After Progression on Prior PD-1 Inhibition	Biological: Durvalumab Radiation: Hypofractionated Radiation Therapy Biological: Tremelimumab	30	Aug 2022
	NCT04547452	Combination of Sintilimab and Stereotactic Body Radiotherapy in AdvancedMetastatic HCC	Radiation: Stereotactic body radiation therapy Drug: Anti-PD-1 antibody drug named Sintilimab	84	July 2022
	NCT03655613	APL-501 or Nivolumab in Combination With APL-101 in Locally Advanced or Metastatic HCC and RCC	Biological: APL-501 Drug: APL-101 Biological: Nivolumab	119	Sept 2020
	NCT04380545	Nivolumab, Fluorouracil, and Interferon Alpha 2B for the Treatment of Unresectable Fibrolamellar Cancer	Drug: Fluorouracil Biological: Nivolumab Biological: Recombinant Interferon Alpha 2b-like Protein	15	July 2021
	NCT02519348	A Study of Durvalumab or Tremelimumab Monotherapy, or Durvalumab in Combination With Tremelimumab or Bevacizumab in Advanced Hepatocellular Carcinoma	Biological: Durvalumab + tremelimumab Biological: Durvalumab Biological: Tremelimumab Biological: Durvalumab + Bevacizumab	433	Nov 2020
	NCT03755739	Trans-Artery/Intra-Tumor Infusion of Checkpoint Inhibitors for Immunotherapy of Advanced Solid Tumors	Drug: Checkpoint inhibitor (CPI) such as Pembrolizumab	200	Nov 2033
	NCT02940496	Pembrolizumab With or Without Elbasvir/Grazoprevir and Ribavirin in Treating Patients With Advanced Refractory Liver Cancer	Drug: Elbasvir/Grazoprevir Other: Laboratory Biomarker Analysis Biological: Pembrolizumab Drug: Ribavirin	30	Dec 2021
	NCT03836352	Study of an Immunotherapeutic, DPX-Survivac, in Combination With Low DoseCyclophosphamide & Pembrolizumab, in Subjects With Selected Advanced & Recurrent Solid Tumors	Other: DPX-Survivac Drug: Cyclophosphamide Drug: Pembrolizumab	184	Dec 2022
	NCT03544723	Safety and Efficacy of p53 Gene Therapy Combined With Immune Checkpoint Inhibitors inSolid Tumors.	Drug: Ad-p53	40	June 2022
	NCT03680508	TSR-022 (Anti-TIM-3 Antibody) and TSR-042 (Anti-PD-1 Antibody) in Patients WithLiver Cancer	Drug: TSR-022 and TSR-042	42	Oct 2022
***CAR-T***	NCT03941626	Autologous CAR-T/TCR-T Cell Immunotherapy for Solid Malignancies	Biological: CAR-T/TCR-T cells immunotherapy	50	Dec 2020
	NCT03638206	Autologous CAR-T/TCR-T Cell Immunotherapy for Malignancies	Biological: CAR-T cell immunotherapy	73	Mar 2023
	NCT03013712	A Clinical Research of CAR T Cells Targeting EpCAM Positive Cancer	Biological: CAR-T cell immunotherapy	60	Dec 2018
***ACT***	NCT03093688	Clinical Safety and Efficacy Study of Infusion of iNKT Cells and CD8+T Cells in Patients With Advanced Solid Tumor	Biological: Infusion of iNKT cells and CD8+T cells	40	Dec 2021
	NCT04502082	Study of ET140203 T Cells in Adults With Advanced Hepatocellular Carcinoma (ARYA-1)	Biological: ET140203 autologous T cell product	50	Jan 2023
	NCT03998033	Study of ET140202 T Cells in Adults With Advanced Hepatocellular Carcinoma	Biological: ET140202 autologous T cell product	50	July 2022
	NCT03592706	Autologous Immune Killer Cells to Treat Liver Cancer Patients as an Adjunct Therapy	Biological: IKC (Immune Killer Cells) Procedure: TACE (Transcatheter Arterial Chemoembolization)	60	Feb 2021
	NCT02856815	Safety and Efficacy of “Immune Cell-LC” in TACE Therapy	Biological: Immuncell-LC	78	October 30, 2020
***OV***	NCT03071094	A Trial to Evaluate the Safety and Efficacy of the Combination of the Oncolytic Immunotherapy Pexa-Vec With the PD-1 Receptor Blocking Antibody Nivolumab in the First-line Treatment of Advanced Hepatocellular Carcinoma (HCC)	Biological: Pexastimogene Devacirepvec (Pexa Vec) Drug: Nivolumab	30	Sept 2020
***Vax***	NCT03067493	RFA Combined With Neo-MASCT for Primary HCC: a Phase II Trial	Biological: Neo-MASCT	98	Mar 2021
**Phase I clinical trials**					
***ICI + combinations***					
Neoadjuvant (Stage A)	NCT03682276	Safety and Bioactivity of Ipilimumab and Nivolumab Combination Prior to Liver Resection in Hepatocellular Carcinoma	Biological: Ipilimumab Biological: Nivolumab	32	Dec 2020
*ICI + TACE (Stage B)*	NCT03143270	A Study to Test the Safety and Feasibility of Nivolumab With Drug Eluting Bead Transarterial Chemoembolization in Patients With Liver Cancer	Drug: Drug Eluting Bead Transarterial Chemoembolization Drug: Nivolumab	14	Apr 2022
*ICI + radioembolization (Stage B)*	NCT03099564	Pembrolizumab Plus Y90 Radioembolization in HCC Subjects	Drug: Pembrolizumab Device: Y90 radioembolization	30	July 2020
	NCT02837029	Nivolumab and Yttrium Y 90 Glass Microspheres in Treating Patients With Advanced Liver Cancer	Other: Laboratory Biomarker Analysis Biological: Nivolumab Radiation: Yttrium Y 90 Glass Microspheres	27	July 2019
	NCT01658878	An Immuno-therapy Study to Evaluate the Effectiveness, Safety and Tolerability of Nivolumab or Nivolumab in Combination With Other Agents in Patients With Advanced Liver Cancer	Biological: Nivolumab Drug: Sorafenib Drug: Ipilimumab Drug: Cabozantinib	1097	Aug 2020
	NCT03474640	Safety, Tolerability and Pharmacokinetics of an Anti-PD-1 Monoclonal Antibody in Subjects With Advanced Malignancies	Biological: Toripalimab, Recombinant Humanized anti-PD-1 Monoclonal Antibody	258	Aug 2022
	NCT03655613	APL-501 or Nivolumab in Combination With APL-101 in Locally Advanced or Metastatic HCC and RCC	Biological: APL-501 Drug: APL-101 Biological: Nivolumab	119	Sept 2020
	NCT04564313	Safety and Efficacy of Camrelizumab (Anti-PD-1 Antibody) in Recurrent HCC After Liver Transplantation	Drug: Camrelizumab treatment	20	July 2021
	NCT02940496	Pembrolizumab With or Without Elbasvir/Grazoprevir and Ribavirin in Treating Patients With Advanced Refractory Liver Cancer	Drug: Elbasvir/Grazoprevir Other: Laboratory Biomarker Analysis Biological: Pembrolizumab Drug: Ribavirin	30	Dec 2021
	NCT03203304	Stereotactic Body Radiotherapy (SBRT) Followed by Immunotherapy in Liver Cancer	Drug: Nivolumab Drug: Ipilimumab	50	Aug 2021
	NCT04220944	Combined Locoregional Treatment With Immunotherapy for Unresectable HCC.	Drug: Sintilimab Procedure: Microwave Ablation Procedure: TACE	45	June 2021
	NCT03864211	Thermal Ablation Followed by Immunotherapy for HCC	Procedure: Thermal ablation Drug: Toriplimab	120	Mar 2021
***New ICI***	NCT04374877	Study of SRF388 in Patients With Advanced Solid Tumors	Drug: SRF388	122	July 2021
***CAR-T***	NCT04121273	GPC3-targeted CAR-T Cell for Treating GPC3 Positive Advanced HCC	Biological: CAR-T cell immunotherapy	20	Oct 2021
	NCT02905188	Glypican 3-specific Chimeric Antigen Receptor Expressing T Cells for Hepatocellular Carcinoma (GLYCAR)	Genetic: GLYCAR T cells Drug: Cytoxan Drug: Fludarabine	14	Dec 2021
	NCT03198546	GPC3-T2-CAR-T Cells for Immunotherapy of Cancer With GPC3 Expression	Biological: GPC3 and/or TGFβ targeting CAR-T cells	30	Aug 2020
	NCT03941626	Autologous CAR-T/TCR-T Cell Immunotherapy for Solid Malignancies	Biological: CAR-T/TCR-T cells immunotherapy	50	Dec 2020
	NCT03638206	Autologous CAR-T/TCR-T Cell Immunotherapy for Malignancies	Biological: CAR-T cell immunotherapy	73	Mar 2023
	NCT03013712	A Clinical Research of CAR T Cells Targeting EpCAM Positive Cancer	Biological: CAR-T cell immunotherapy	60	Dec 2018
***ACT***	NCT03093688	Clinical Safety and Efficacy Study of Infusion of iNKT Cells and CD8+T Cells in Patients With Advanced Solid Tumor	Biological: Infusion of iNKT cells and CD8+T cells	40	Dec 2021
	NCT04032392	Immunotherapy of Advanced Hepatitis B Related Hepatocellular Carcinoma With γδ T Cells	Biological: autologous γδ T cells	20	July 2021
	NCT04502082	Study of ET140203 T Cells in Adults With Advanced Hepatocellular Carcinoma (ARYA-1)	Biological: ET140203 autologous T cell product	50	Jan 2023
	NCT03998033	Study of ET140202 T Cells in Adults With Advanced Hepatocellular Carcinoma	Biological: ET140202 autologous T cell product	50	July 2022
	NCT03132792	AFP^c332^T in Advanced HCC	Genetic: Autologous genetically modified AFP^c332^T cells	45	June 2021
	NCT03441100	TCR-engineered T Cells in Solid Tumors: IMA202-101	Drug: IMA202 Product Device: IMA_Detect	15	June 2022
	NCT03319459	FATE-NK100 as Monotherapy and in Combination With Monoclonal Antibody in Subjects With Advanced Solid Tumors	Drug: FATE-NK100 Drug: Cetuximab Drug: Trastuzumab	100	Oct 2021
	NCT03841110	FT500 as Monotherapy and in Combination With Immune Checkpoint Inhibitors in Subjects With Advanced Solid Tumors	Drug: FT500 Drug: Nivolumab Drug: Pembrolizumab (and 3 more…)	76	Mar 2022
***Agonists/Cytokines***	NCT02315066	Study Of OX40 Agonist PF-04518600 Alone And In Combination With 4-1BB Agonist PF-05082566	Drug: PF-04518600 Drug: PF-04518600 plus PF-05082566	176	Dec 2020
	NCT03655002	IRX-2, Cyclophosphamide, and Nivolumab in Treating Patients With Recurrent or Metastatic and Refractory Liver Cancer	Drug: Cyclophosphamide Biological: Cytokine-based Biologic Agent IRX-2 Biological: Nivolumab	28	June 2022
***OV***	NCT03071094	A Trial to Evaluate the Safety and Efficacy of the Combination of the Oncolytic Immunotherapy Pexa-Vec With the PD-1 Receptor Blocking Antibody Nivolumab in the First-line Treatment of Advanced Hepatocellular Carcinoma (HCC)	Biological: Pexastimogene Devacirepvec (Pexa Vec) Drug: Nivolumab	30	Sept 2020
***Vax***	NCT04248569	DNAJB1-PRKACA Fusion Kinase Peptide Vaccine Combined With Nivolumab and Ipilimumab for Patients With Fibrolamellar Hepatocellular Carcinoma	Drug: DNAJB1-PRKACA peptide vaccine Drug: Nivolumab Drug: Ipilimumab	12	Mar 2024

## The Future of Immunotherapies in HCC Beyond ICI

**Figure 6:**
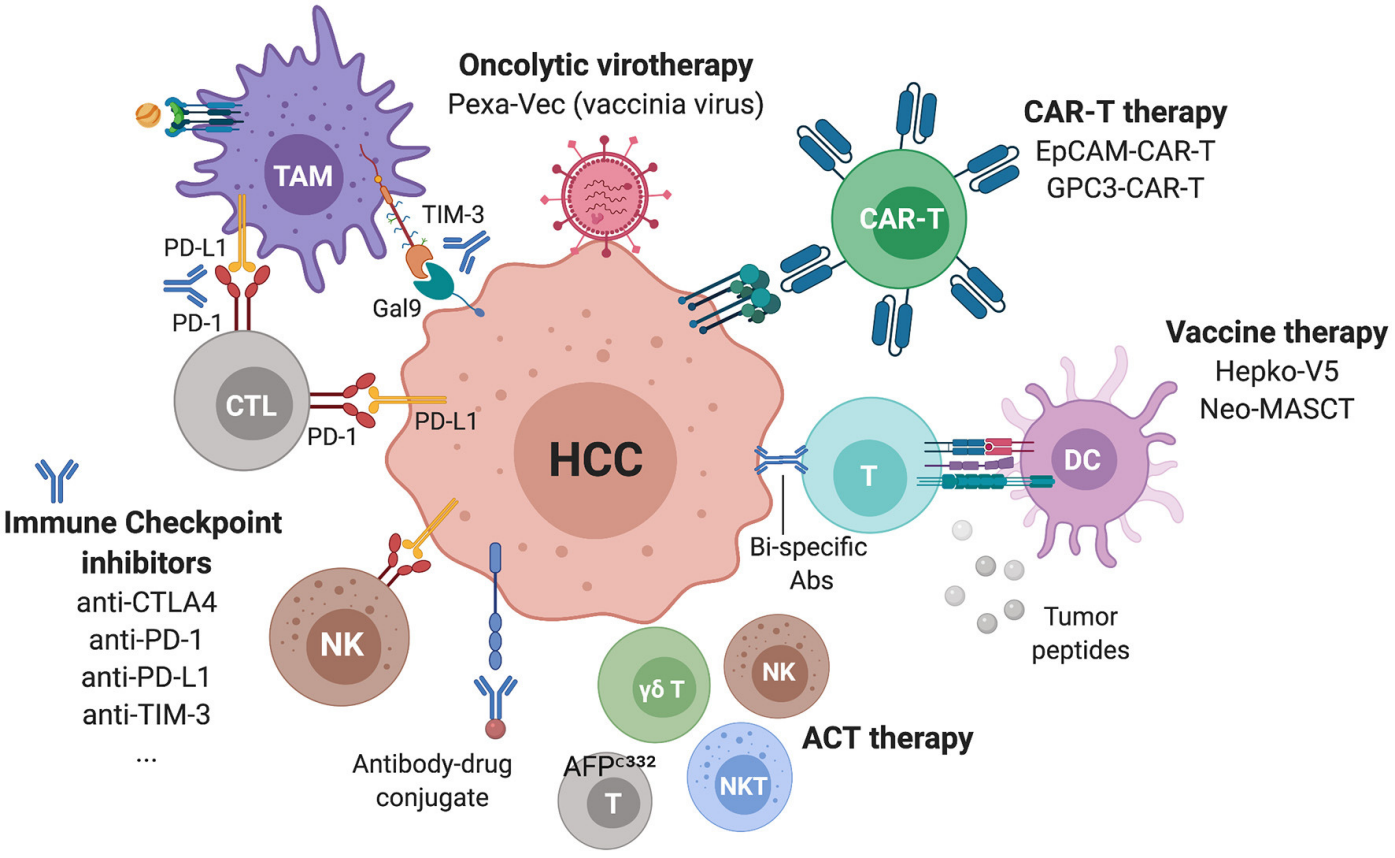
Immunotherapies in ongoing clinical trials for advanced HCC. Several ICIs targeting checkpoints on lymphocytes but also NK and myeloid cells are currently being assessed as monotherapies or in combinations. Additional strategies include CAR-T cells, oncolytic viruses, vaccines, antibody-drug conjugates and bi-specific antibodies.

Besides ICI, several immunotherapeutic strategies for HCC patients are emerging, such as targeted therapies promoting ADCC, adoptive cell therapy (ACT), including the transfer of autologous CD8 T cells, iNKT cells, γδ T cells, cytokine-induced immune killer cells (IKC), chimeric antigen receptor (CAR)-T cells, oncolytic viruses and vaccines (Figure 6). For a number of these strategies, tumor-specific antigens (TSA) or tumor-associated antigens (TAA) are targeted. In HCC, these include α-fetoprotein (109–111), hTERT (112), glypican-3 (GPC3) (113–115), p53 (116), melanoma antigen gene A (MAGE-A) (117), squamous cell carcinoma antigen recognized by T cells (SART) (118), and NY-ESO-1 (119). More recently, the oncogenic phosphatase PRL3 was confirmed as a TAA, as it was shown to be expressed in tumors, but not in patient-matched normal tissue, across 11 cancers. A humanized antibody targeting this TAA, PRL3-zumab, was shown to enhance the intra-tumoral recruitment of B cells, NK cells and macrophages, suggesting that this antibody might promote tumor killing by ADCC (120). Similarly, the elevated expression of GPC3 in >70% of HCC, and its association with poor prognosis (121), has led to the development of several immunotherapeutic strategies, including the humanized monoclonal antibody codrituzumab (122), bi-specific antibodies (123), CAR-T cells (124), antibody-drug conjugates (125), and vaccines (126). GPC3-CAR-T cells have been shown to be polyfunctional and capable of eliminating HCC in a transplantable orthotopic mouse model (127), and there are currently at least 5 phase I clinical trials recruiting patients with HCC to test GPC3-CAR-T cells (Table 1; ClinicalTrials.gov, December 2020). Another CAR-T cell tested in multiple solid tumors including HCC is the EpCAM-CAR-T, as registered in a phase I/II trial (NCT03013712). In a similar approach, a phase I trial is testing the transfer of autologous genetically modified AFP^c332^T cells, T cells expressing an enhanced TCR Specific for α-fetoprotein in HLA-A2 positive patients with advanced HCC (NCT03132792). In the oncolytic viruses sphere, a phase III trial (NCT02562755) is testing a vaccinia virus-based immunotherapy, Pexastimogene Devacirepvec (Pexa-Vec), in patients with advanced HCC, based on promising results from the phase IIb trial TRAVERSE (128). Pexa-Vec is also being tested in combination with nivolumab in the first-line treatment of advanced HCC in a phase II trial (NCT03071094). As for vaccines, a phase III trial (NCT02232490) is evaluating the benefit of hepcortespenlisimut-L (Hepko-V5), an oral allogeneic vaccine derived from patients' blood, given in an experimental arm vs. placebo, to patients with advanced HCC. Similarly, a phase II trial (NCT03067493) is testing the Neoantigen Multiple Target Antigen Stimulating Cell Therapy (Neo-MASCT) vaccine, which consists of 18 cycles, each including one DC subcutaneous injection and one CTL infusion.

## Conclusions

HCC comprises a heterogeneous set of cancers with different etiologies, mutations and immune microenvironments, as demonstrated by broad molecular and immunological classifications. The advent of recent technologies including single cell approaches is now allowing high resolution characterization of the immune landscapes of HCC and is expected to uncover novel immunotherapeutic targets and approaches tailored to patients. ICI combination therapies are expected to dramatically improve the systemic therapy of advanced HCC. However, the prioritization of different combinations requires additional understanding of liver-specific immunity and the validation of therapeutic targets in suitable pre-clinical models of HCC taking into consideration the genetic heterogeneity of tumor cells and the cirrhotic or NASH environments. Further, an in-depth characterization of the biomarkers leading to improved patients' response to the various such combinations will contribute to better selection of patients and ameliorate the outcome. Last, a critical issue not discussed here is the management of immune-related adverse events (irAEs) often elicited by immunotherapies and that should be considered in designing and implementing immunotherapies. It is hoped that with the rapidly evolving field of oncoimmunology and trials in different cancer types, we will learn valuable lessons for future drug discovery in HCC.

## Author Contributions

JG and MS conceived the structure of this review. DC contributed to the description of the immune landscape of the liver and of HCC and prepared Figure 3. J-FB contributed to the review of clinical activity in HCC. All authors revised the manuscript and approved the final version.

## Conflict of Interest

The authors declare that the research was conducted in the absence of any commercial or financial relationships that could be construed as a potential conflict of interest.

## Abbreviations

ACT: adoptive cell therapy
ADCC: antibody-dependent cell cytotoxicity
BCLC: Barcelona Clinic Liver Cancer
CAR: chimeric antigen receptors
DAMPs: danger-associated molecular patterns
EC: endothelial cells
ECM: extracellular matrix
EMT: epithelial-to-mesenchymal transition
fDC: follicular dendritic cell
HBV: hepatitis B virus
HCC: hepatocellular carcinoma
HCV: hepatitis C virus
HSC: hepatic stellate cells
ICI: immune checkpoint inhibitor
IFNg: interferon gamma
IHC: immunohistochemistry
ILCs: innate lymphoid cells
IKC: immune killer cells
irAEs: immune-related adverse events
KC: Kupffer cells
LIHC: liver hepatocellular carcinoma
LSEC: liver sinusoidal endothelial cells
MAGE-A: melanoma antigen gene A
MAMPs: microbial-associated molecular patterns
MAPK: mitogen-activated protein kinase
MDSCs: myeloid-derived suppressor cells
MNPs: mononuclear phagocytes
mOS: median overall survival
mregDCs: mature DCs enriched in immunoregulatory molecules
mTOR: mammalian target of rapamycin
NK cells: natural killer cells
cNK cells: conventional NK cells
LrNK cells: liver-resident NK cells
NKT cells: natural killer T cells
NSCLC: non-small cell lung cancer
ORR: overall response rate
pDC: plasmacytoïd dentritic cells
PFS: progression-free survival
PI(3)K: Phosphoinositide 3-kinase
RTK: receptor tyrosine kinase
SART: squamous cell carcinoma antigen recognized by T cells
scRNAseq: single cell RNA sequencing
SNPs: single nucleotide polymorphisms
TAA: tumor-associated antigens
TACE: transarterial chemo-embolization
TAMs: tumor-associated macrophages
TCR: T-cell receptor
Tex: exhausted T cells
TIC: tumor-initiating cells
TILs: tumor-infiltrating lymphocytes
TKi: tyrosine kinase inhibitors
TLS: tertiary lymphoid structures
TME: tumor microenvironment
Treg: regulatory T cells
TSA: tumor-specific antigens.
