# Supplementary material for: Hepatocellular Carcinoma Immune Landscape and the Potential of Immunotherapies
Source: Front Immunol. 2021 Mar 18;12:655697. doi: 10.3389/fimmu.2021.655697 (PMC8012774; doi:10.3389/fimmu.2021.655697)
Supplement: Supplementary file 1 [file Table_1.docx]

**Supplementary Table 1**

| Gene symbol | Protein symbol | Protein name |
| --- | --- | --- |
| *ACTA2*  *ACKR1*  *ALB*  *ALDH1A1*  *ANPEP*  *APOE*  *ARID1A*  *ARID2*  *ATP7B*  *B7S1R*  *C1QA*  *C1QB*  *CADM1*  *CCL2*  *CCL3*  *CCL5*  *CCL11*  *CCL19*  *CCND1*  *CCR1*  *CCR3*  *CCR7*  *CD1D*  *CD1E*  *CDH5*  *CDKN2A*  *CFTR*  *CLEC4C*  *CLEC4G*  *CLEC4M*  *CLEC9A*  *CLEC10A*  *COL1A1*  *CPA3*  *CSF1*  *CSF1R*  *CTLA4*  *CTNNB1*  *CX3CR1*  *CXCL16*  *CXCR6*  *DCN*  *DPP4*  *ENG*  *EOMES*  *EPCAM*  *FAH*  *FAP*  *FCER1A*  *FCN1*  *FGF19*  *FGFBP2*  *FOLR2*  *FOXP3*  *G6PC*  *GNLY*  *GPC3*  *GZMA*  *GZMB*  *HAVCR2*  *HFE*  *HIF1A*  *HLA-DRA*  *ICAM1*  *IGHA1*  *IGHG3*  *IGHGP*  *IL6*  *IL10*  *IL13*  *IL33*  *IL7R*  *KDR*  *KEAP1*  *KLRC1*  *KLRD1*  *KLRF1*  *KLRK1*  *KRT19*  *LAG3*  *LAMP3*  *LAYN*  *LILRA4*  *LILRB5*  *LTB*  *LYZ*  *MARCO*  *MET*  *MKI67*  *MNDA*  *MS4A1*  *MYC*  *MZB1*  *NCR1*  *NCR3*  *NFE2L2*  *PDCD1*  *PDGFA*  *PDGFRA*  *PDL1*  *PDPN*  *PECAM1*  *PIK3CA*  *PLK1*  *PLVAP*  *PROM1*  *PRF1*  *RGS5*  *RPS6KA2*  *S100A12*  *SOX9*  *SPON2*  *SPTBN1*  *SPP1*  *STMN*  *TACSTD2*  *TBX21*  *TCF1A*  *TCL1A*  *TERT*  *TGFB1*  *THBS1*  *TIGIT*  *TIMD4*  *TNFRSF12A*  *TOX*  *TP53*  *TPSAB1*  *TPSB2*  *TRDC*  *TREM1*  *TREM2*  *VCAN*  *VEGFA*  *VIM*  *VWA1*  *VWF*  *XCL1*  *XCL2*  *YAP1* | ACTA2  ACKR1 – CD234  ALB  ALDH1A1  ANPEP  APOE  ARID1A  ARID2  ATP7B  B7S1R  C1QA  C1QB  CADM1  CCL2  CCL3  CCL5  CCL11  CCL19  CCND1  CCR1  CCR3  CCR7  CD1d  CD1e  CDH5  CDKN2A  CFTR  CLEC4C - CD303  CLEC4G – CD370  CLEC4M – CD299  CLEC9A  CLEC10A  COL1A1  CPA3  CSF1  CSF1R  CTLA4 - CD152  β-catenin  CX3CR1  CXCL16  CXCR6 - CD186  DCN  DPP4 - CD26  ENG  EOMES  EPCAM  FAH  FAP  FCER1A  FCN1  FGF19  FGFBP2  FOLR2  FOXP3  G6PC1  GNLY  GPC3  GZMA  GZMB  TIM3  HFE  HIF1α  HLA-DRA  ICAM1 - CD54  IGHA1  IGHG3  IGHGP  IL-6  IL-10  IL-13  IL-33  IL-7R  VEGF-R2  KEAP1  NKG2A  KLRD1  KLRF1  NKG2D  CK19  LAG3 - CD223  LAMP3  LAYN  LILRA4  LILRB5 - CD85  LTB  LYZ  MARCO  MET  Ki67  MNDA  MS4A1  MYC  MZB1  NCR1  NCR3  NFE2L2  PD-1 - CD278  PDGFα  PDGFRA  PD-L1 - CD274  PDPN  PECAM1  PI3Kα  PLK1  PLVAP  CD133  PRF1  RGS5  RSK3  S100A12  SOX9  SPON2  SPTBN1  OSM  STMN  TROP2  T-bet  TCF1  TCL1A  TERT  TGF-β  THBS1  TIGIT  TIMD4  TNFRSF12A  TOX  p53  TRYB1  TRYB2  TRDC  TREM1  TREM2  VCAN  VEGF  VIM  VWA1  VWF  XCL1  XCL2  YAP | Actin, aortic smooth muscle  Atypical chemokine receptor 1  Albumin  A[ldehyde dehydrogenase 1 family member A1](https://www.genenames.org/data/gene-symbol-report/#!/hgnc_id/HGNC:402)  A[minopeptidase M](https://www.genenames.org/data/gene-symbol-report/#!/hgnc_id/HGNC:500)  Apolipoprotein E  AT-rich interactive domain-containing protein 1A  AT-rich interactive domain-containing protein 2  [ATPase copper transporting beta](https://www.genenames.org/data/gene-symbol-report/#!/hgnc_id/HGNC:870)  B7 superfamily member1  Complement C1q subcomponent subunit A  Complement C1q subcomponent subunit B  Cell adhesion molecule 1  C-C motif chemokine ligand 2  C-C motif chemokine ligand 3  C-C motif chemokine ligand 5  C-C motif chemokine ligand 11  C-C motif chemokine ligand 19  Cyclin D1  C-C motif chemokine receptor 1  C-C motif chemokine receptor 3  C-C motif chemokine receptor 7  CD1d molecule  CD1e molecule  Cadherin 5  Cyclin dependent kinase inhibitor 2A  CF transmembrane conductance regulator cytotoxic  C-type lectin domain family 4 member C  C-type lectin domain family 4 member G  C-type lectin domain family 4 member M  C-type lectin domain containing 9A  C-type lectin domain family 10 member A  Collagen type I alpha 1 chain  Carboxypeptidase A3  Colony stimulating factor 1  Colony stimulating factor 1 receptor  T-lymphocyte-associated protein 4  Catenin beta-1  C-X3-C motif chemokine receptor 1  C-X-C motif chemokine ligand 16  C-X-C motif chemokine receptor 6  Decorin  Dipeptidyl peptidase 4  Endoglin  Eomesodermin  Epithelial cell adhesion molecule  Fumarylacetoacetate  Fibroblast activation protein alpha  Fc fragment of IgE receptor 1A  Ficolin 1  Fibroblast growth factor 19  Fibroblast growth factor binding protein 2  Folate receptor beta  Forkhead box P3  Glucose-6-phosphatase catalytic subunit 1  Granulysin  Glypican 3  Granzyme A  Granzyme B  Hepatitis A virus cellular receptor 2  Homeostatic iron regulator  Hypoxia-inducible factor 1-alpha  Major histocompatibility complex, class II, DR alpha  Intercellular adhesion molecule  Immunoglobulin heavy constant alpha 1  Immunoglobulin heavy constant gamma 3  Immunoglobulin heavy constant gamma P  Interleukin-6  Interleukin-10  Interleukin-13  Interleukin-33  Interleukin 7 receptor  Vascular endothelial growth factor receptor type 2  Kelch-like ECH-associated protein 1  Killer cell lectin like receptor C1  Killer cell lectin like receptor D1  Killer cell lectin like receptor F1  Killer cell lectin like receptor K1  Cytokeratin 19  [Lymphocyte activating 3](https://www.genenames.org/data/gene-symbol-report/#!/hgnc_id/HGNC:6476)  Lysosomal associated membrane protein 3  Layilin  Leukocyte immunoglobulin like receptor A4  Leukocyte immunoglobulin like receptor B5  Lymphotoxin beta  Lysozyme  Macrophage receptor with collagenous structure  MET Proto-Oncogene, receptor tyrosine kinase  Marker of proliferation Ki-67  Myeloid cell nuclear differentiation antigen  Membrane spanning 4-domains A1  MYC proto-oncogene, BHLH transcription factor  Marginal zone B and B1 cell specific protein  Natural cytotoxicity triggering receptor 1  Natural cytotoxicity triggering receptor 3  Nuclear factor, erythroid 2 like 2  Programmed cell death 1  Platelet derived growth factor subunit A  Platelet derived growth factor receptor alpha  Programmed cell death 1 ligand 1  Podoplanin  Platelet and endothelial cell adhesion molecule 1  Phosphatidylinositol-4,5-bisphosphate 3-kinase catalytic subunit alpha  Polo like kinase 1  Plasmalemma vesicle associated protein  Prominin 1  Perforin 1  Regulator of G protein signaling 5  Ribosomal protein S6 kinase A2  S100 Calcium Binding Protein A12  SRY-Box transcription factor 9  Spondin 2  Spectrin Beta, non-erythrocytic 1  Osteopontin  Stathmin 1  Tumor associated calcium signal transducer 2  T-box transcription factor 21  HMG-box transcription factor Tcf-1  TCL1 Family AKT Coactivator A  Telomerase reverse transcriptase  Transforming growth factor beta  Thrombospondin 1  T cell immunoreceptor with Ig and ITIM domains  T cell immunoglobulin and mucin domain containing 4  TNF receptor superfamily member 12A  Thymocyte selection associated high mobility group box  Tumor protein P53  Tryptase alpha/beta 1  Tryptase beta 2  T cell receptor delta constant  Triggering receptor expressed on myeloid cells 1  Triggering receptor expressed on myeloid cells 2  Versican  Vascular endothelial growth factor  Vimentin  von Willebrand factor A domain-containing protein 1  von Willebrand factor protein  X-C motif chemokine ligand 1  X-C motif chemokine ligand 2  Yes-associated protein |
